# Terebra steering in chalcidoid wasps

**DOI:** 10.1186/s12983-023-00503-1

**Published:** 2023-08-08

**Authors:** Benjamin Eggs, Stefan Fischer, Michael Csader, István Mikó, Alexander Rack, Oliver Betz

**Affiliations:** 1 Evolutionary Biology of Invertebrates, Institute of Evolution and Ecology, University of Tübingen, Auf der Morgenstelle 28, 72076 Tübingen, Germany; 2 Tübingen Structural Microscopy Core Facility (TSM), University of Tübingen, Schnarrenbergstraße 94–96, 72076 Tübingen, Germany; 3 State Museum of Natural History Karlsruhe, Erbprinzenstraße 13, 76133 Karlsruhe, Germany; 4 Department of Biological Sciences, University of New Hampshire Collection of Insects and Other Arthropods, University of New Hampshire, Spaulding Hall, Durham, NH 03824 USA; 5 ESRF – The European Synchrotron, Structure of Materials Group – ID19, CS 40220, 38043 Grenoble Cedex 9, France

**Keywords:** Chalcidoidea, Functional morphology, Hymenoptera, Ovipositor, Parasitoid, Pteromalidae

## Abstract

Various chalcidoid wasps can actively steer their terebra (= ovipositor shaft) in diverse directions, despite the lack of terebral intrinsic musculature. To investigate the mechanisms of these bending and rotational movements, we combined microscopical and microtomographical techniques, together with videography, to analyse the musculoskeletal ovipositor system of the ectoparasitoid pteromalid wasp *Lariophagus distinguendus* (Förster, 1841) and the employment of its terebra during oviposition. The ovipositor consists of three pairs of valvulae, two pairs of valvifers and the female T9 (9th abdominal tergum). The paired 1st and the 2nd valvulae are interlocked via the olistheter system, which allows the three parts to slide longitudinally relative to each other, and form the terebra. The various ovipositor movements are actuated by a set of nine paired muscles, three of which (i.e. 1st valvifer-genital membrane muscle, ventral 2nd valvifer-venom gland reservoir muscle, T9-genital membrane muscle) are described here for the first time in chalcidoids. The anterior and posterior 2nd valvifer-2nd valvula muscles are adapted in function. (1) In the active probing position, they enable the wasps to pull the base of each of the longitudinally split and asymmetrically overlapping halves of the 2nd valvula that are fused at the apex dorsally, thus enabling lateral bending of the terebra. Concurrently, the 1st valvulae can be pro- and retracted regardless of this bending. (2) These muscles can also rotate the 2nd valvula and therefore the whole terebra at the basal articulation, allowing bending in various directions. The position of the terebra is anchored at the puncture site in hard substrates (in which drilling is extremely energy- and time-consuming). A freely steerable terebra increases the chance of contacting a potential host within a concealed cavity. The evolution of the ability actively to steer the terebra can be considered a key innovation that has putatively contributed to the acquisition of new hosts to a parasitoid’s host range. Such shifts in host exploitation, each followed by rapid radiations, have probably aided the evolutionary success of Chalcidoidea (with more than 500,000 species estimated).

**Supplementary Information:**

The online version contains supplementary material available at 10.1186/s12983-023-00503-1.

## Background

The evolution of parasitoidism in Hymenoptera has led to one of the largest species radiations in insects [1–5]. A large proportion of parasitoids belong to the Chalcidoidea, an extremely diverse and ecologically important group (nearly 27,000 species described, over 500,000 species estimated) of mainly minute wasps (average body size range from 1–2 mm) that are omnipresent in almost all terrestrial habitats [6–11]. Most chalcidoids are ectoparasitoids of other insects, usually attacking enclosed host stages with reduced mobility (i.e. egg or larval stages of wood and stem borers, leaf-miners or inhabitants of galls, seeds and fruits) [12], although other life stages are also targeted [13]. The parasitization of hosts living deep within substrates allows the ectoparasitoid larvae to develop within the protection of a concealed environment and without exposure to the host immune system as occurs in endoparasitoids. An evolutionary novelty and presumably a strong driver of diversification in Chalcidoidea is the secondary reversal to monocondylic mandibles (reduction of the posterior condyle accompanied with modified musculature with functional separation), which allow the emerging wasp to bite through a hard substrate by precise cutting movements that overcome the limitations of a single degree of freedom [14]. However, the use of hosts living concealed within hard substrates poses challenges not only for the emerging wasp (i.e. in leaving the substrate), but also for females attempting to parasitize them (i.e. entering the substrate to find a potential host) [15]. In this context, the ovipositor has to fulfil several functional demands: penetration or navigation through the substrate or the target egg/puparium, assessment of the host, discrimination between suitable and previously parasitized hosts, piercing of the host, injection of venom, formation of a feeding tube for host feeding, ovicide or larvicide of the competitors’ eggs or larvae, respectively, marking of the host and find a suitable place for egg laying and oviposition [16]. However, putative evolutionary novelties of the chalcidoid ovipositor system, such as morphological and behavioural adaptations that enable the steering of the terebra (= ovipositor (shaft) sensu [17–27]) and its underlying mechanisms have not been thoroughly investigated hitherto.

As in all hymenopterans, the chalcidoid ovipositor consists of the female T9 (9th abdominal tergum; = outer ovipositor plates sensu [17–25]), two pairs of valvifers and three pairs of valvulae derived from the 8th and 9th abdominal segments (7th and 8th metasomal segments). The basally situated valvifers accommodate the operating musculature, whereas all the valvulae are devoid of intrinsic musculature [28–32]. The 1st valvifers (8th gonocoxites [33, 34] or the fusion of the same with the gonangula [30]; = fulcral plates sensu [17–25, 35–37]; = gonangulum, gonangula sensu [26, 27]) are anteriorly continuous with the rami of the 1st valvulae (8th gonapophyses; = stylets sensu [17–25, 35–38]; = lower valves sensu [26, 27]), and their posterior angles articulate dorsally with the female T9 via the tergo-valvifer articulation and ventrally with the 2nd valvifers via the intervalvifer articulation. The 2nd valvifers (9th gonocoxites; = inner ovipositor plates sensu [17–25]) extend as the 3rd valvulae (9th gonostyli; = (articulating/terminal) palps sensu [19, 20, 22, 23, 36]; = ovipositor sheaths sensu [26, 27]) and are ventrally articulated with the 2nd valvula (fusion of the 9th gonapophyses; = (stylet) sheath sensu [17–25, 36–38]; = upper valve sensu [26, 27]) [28, 29], which is asymmetrically split except at the apex in all chalcidoid families [39]. The two overlapping asymmetric halves of the 2nd valvula are connected dorsally by the notal membrane, which extends almost to the apex [17, 19–25, 40]. The interlocked 1st and 2nd valvulae enclose the egg canal and form the terebra, which is embraced by the 3rd valvulae when not in use. The ventral surface of the 2nd valvula is interlocked with both of the 1st valvulae by a sublateral longitudinal tongue called the rhachis, which runs within a corresponding groove called the aulax along the dorsal surface of each of the 1st valvulae. This so-called olistheter system allows the three elements of the terebra to slide longitudinally relative to each other and simultaneously prevents their unwanted separation [29, 39].

In order to reach their hosts and permit greater control over egg placement, several parasitoid wasps are able actively to bend and rotate their terebra in any direction relative to their body axis [41–44], despite the lack of intrinsic terebral musculature. Such terebra movements have also been reported in chalcidoid wasps of the family of Pteromalidae [40, 45–47], a polyphyletic group sensu lato [6, 10, 48, 49] (over 3500 species described [8]). However, little is known about the actuation of the various ovipositor movements, with the mechanisms involved in terebra steering (i.e. bending and rotating) remaining unclear. In this study, we investigated the working mechanisms of the terebra steering movements of *Lariophagus distinguendus* (Förster, 1841) (Chalcidoidea: Pteromalidae: Pteromalinae), a cosmopolitan synanthropic synovigenic autogenous solitary idiobiont larval and pupal ectoparasitoid of several granivorous coleopteran species [50, 51]. This species exhibits extensive terebra movements during the assessment of a potential host and eventual subsequent egg placement [45, 47]. We aimed (1) to analyse the oviposition process in vivo, (2) to describe the ovipositor of *L. distinguendus*, including all inherent cuticular elements and muscles, (3) to examine the mechanics and mode of function of the musculoskeletal system, including the actuation of the various ovipositor movements, (4) to investigate the underlying working mechanisms of the terebra steering movements and (5) to discuss their eco-evolutionary significance.

## Results and discussion

**Fig. 1:**
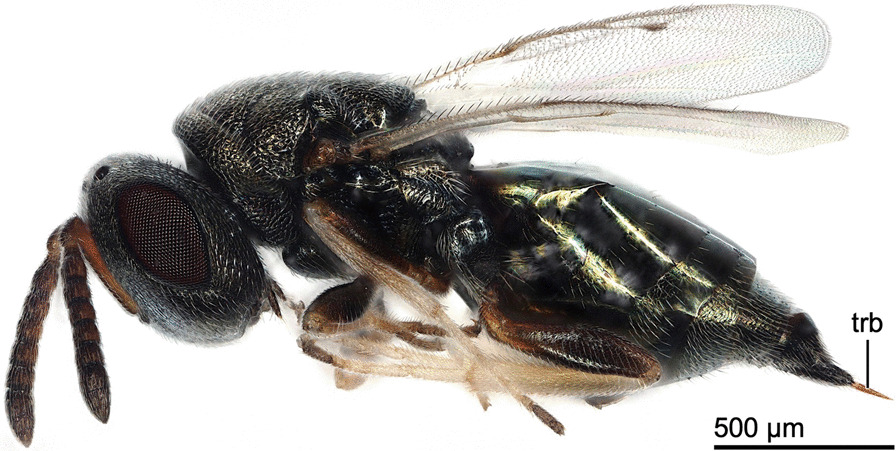
Habitus image of a female *Lariophagus distinguendus* (lateral view). Abbreviations: trb: Terebra

We combined behavioural analyses involving high-resolution video recordings with morphological investigations based on microscopical and microtomographical techniques. These studies have enabled us to present a thorough morphological and mechanical analysis of the musculoskeletal ovipositor system that steers the various movements executed by the female *L. distinguendus* (Fig. 1) during oviposition. In particular, we focused on the employment of the terebra and on its form, structure and material properties.

Morphological terms are applied according to the Hymenoptera Anatomy Ontology (HAO; [52–54]; a table of all 210 terms relevant to the hymenopteran ovipositor system, their definitions and 513 synonyms commonly found in literature is given in Table 2 in the Appendix 1).

In cases in which our findings have been confirmed by other studies, these are indicated below with ‘cf.’.

### Oviposition process and employment of the terebra

**Fig. 2:**
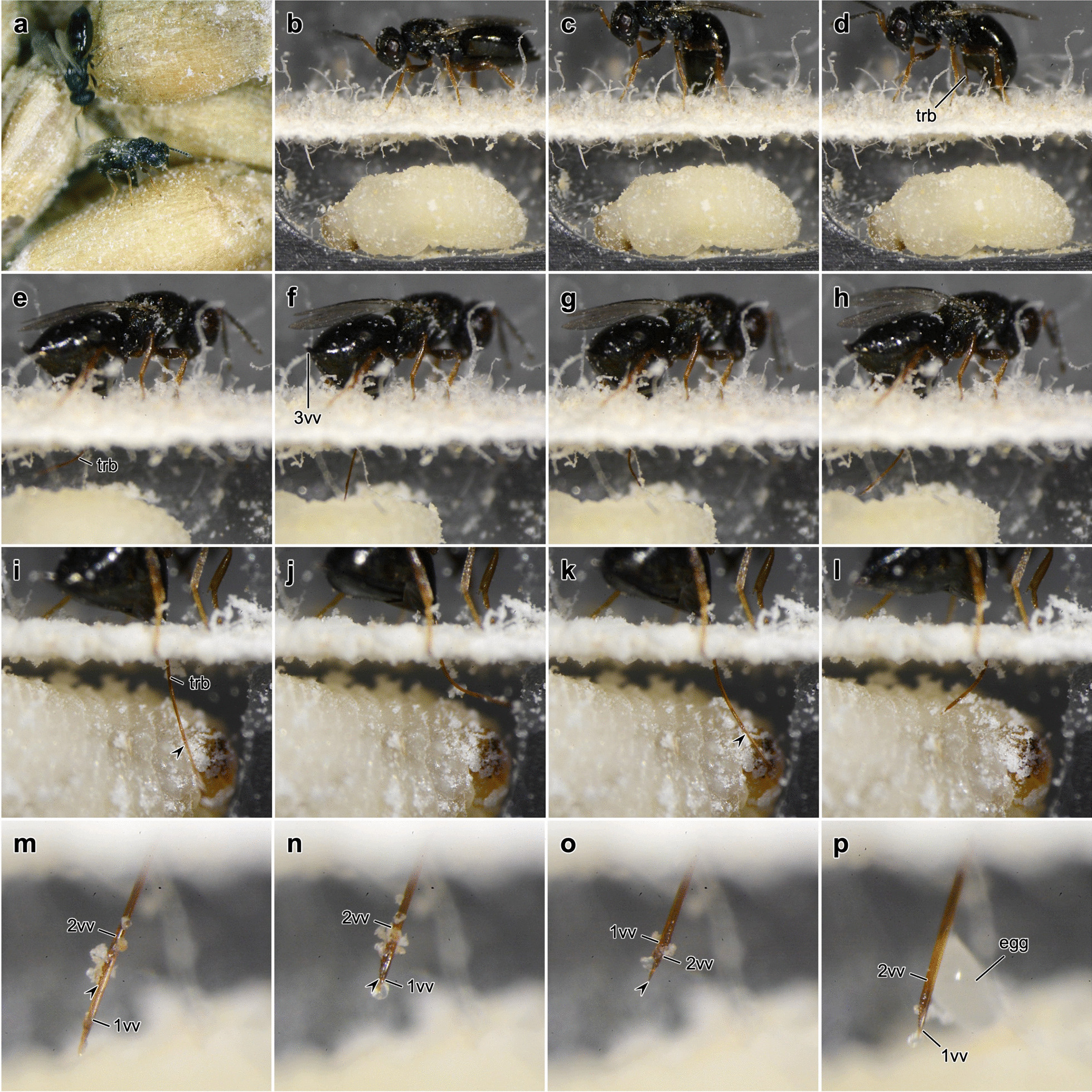
Oviposition process of *Lariophagus distinguendus*. **a** Female wasps search for potential hosts that live in grains of the common wheat *Triticum aestivum*. **b**–**p** Single frames of high-resolution video recordings of a female *L. distinguendus* parasitizing a larva of *Sitophilus granaries* in an artificial chamber (cf. Additional file 1). The wasp and the beetle larva were separated by a piece of blotting paper. After the wasp finds a host (**b**), it brings its terebra into drilling position by a downward bending of the metasoma (**c**) and then, once the apex of the terebra is engaged in the substrate, it lifts the metasoma and the 3rd valvulae upwards (**d**). Following penetration of the substrate, the wasp permanently paralyzes the host larva by venom injection and then usually forms a feeding tube for host feeding. During the subsequent assessment of the host and the search for a suitable place for oviposition, the wasp is able to actively bend (**e**–**l**) its terebra in various directions and also to rotate it to a certain degree (**o**). The individual movements of the single valvulae can be observed (**m**–**p**). The 1st valvulae is frequently protracted far beyond the apex of the 2nd valvula (marked with an arrowhead in **i**, **k**, **m**, **n**, **o**). Finally, an egg is laid (**p**). Rapid alternating movements of the 1st valvulae can be observed during substrate drilling, host envenomation and egg laying. Abbreviations: 1vv: 1st valvula; 2vv: 2nd valvula; 3vv: 3rd valvula; trb: Terebra

Previous studies describing the behavioural sequences of the attempts of *L. distinguendus* [45, 50, 55, 56] and other pteromalids [40, 46, 57] to oviposit have been unable to provide an analysis of the events that take place within the cavity of the substrate. Therefore, we mainly focus on the employment of the terebra and its movements in the following (Fig. 2; Additional file 1).

**Search for the host's habitat**: *L. distinguendus* parasitizes concealed granivorous host larvae (Fig. 2a). The parasitoids mainly use volatile chemicals to locate the habitat of their hosts: faecal cues from the host itself and herbivory-associated chemicals in the seed induced by the mechanical damage caused by the host larvae [51, 55, 58].

**Search for an infested substrate**: Once *L. distinguendus* finds the host’s location (infested grains; blotting paper with the host faeces in our experimental setup), the wasp starts to walk on the substrate followed by antennal drumming with the flagellum directed towards the ground (Additional file 1, min. 0:05–0:07; cf. [45, 47, 50, 55, 56]). The female parasitoid is able to discriminate between healthy and infested grains [59].

**Penetration of the substrate**: Once the female wasp has selected a small spot with its antennae, it brings its terebra into the drilling position by a downward bending of the metasoma so that its tip taps the surface. The terebra is guided and stabilized by the 3rd valvulae in order to prevent buckling despite axial compressive forces occurring during the initial puncturing of the substrate, i.e. the pericarp of the grain. Once the apex of the terebra is engaged in the substrate, the metasoma with the 2nd valvifer and the attached 3rd valvulae are lifted upwards out of the way (Fig. 2b–d; Additional file 1, min. 0:06–0:11; cf. [40, 45, 47, 50, 55, 56]). The initial puncturing (i.e. pericarp surface penetration) is necessary for the 1st and 2nd valvulae to be anchored in the substrate so that the subsequent ‘push-pull’ mechanism can be initiated. Thereby, the wasp exhibits alternate reciprocal movements of the paired 1st valvulae, which can be seen as trembling movements of the posteroventral part of the metasoma (i.e. the 2nd valvifers and the female T9). Only one of the 1st valvulae is pushed into the substrate at a time, while the other 1st valvula and the 2nd valvula, which are anchored in the substrate, are simultaneously pulled [60–62]. The apical sawteeth thereby increase the friction with the surrounding substrate. The tension in the two anchored ‘stationaryʼ elements increases their bending stiffness and, hence, they can serve as guides for the particular 1st valvula being pushed into the substrate [44, 60]. Small pushing movements of the 2nd valvula caused by the relative movements of the 2nd valvifers cannot be excluded (cf. [32]). The simultaneous pushing and pulling of the various terebral elements minimizes the net compressive force on the substrate and thus the chance of buckling of the terebra [32, 44, 60]. The ‘push-pull’ mechanism enables drilling without torque and with very low axial load, although these cannot be completely avoided [44, 62]. During the drilling process (Additional file 1, min. 0:12–0:20; cf. [47]), the wasp combines the ‘push-pull’ mechanism with slight rotations of the terebra [44, 60]. Moreover, a fluid is constantly secreted at the apex and also along the shaft of the terebra. This secretion putatively prevents particles from entering the terebra but might also act as a (cooling) lubricant (cf. [63, 64]).

**Search for a potential host within the substrate**: As soon as the wasp has penetrated the grain in which a potential host larva is living, it attempts to locate the host larva in its concealed cavity with its terebra (Additional file 1, min. 4:02–4:32). Thereby, the metasoma is frequently rotated by up to 35° from the longitudinal body axis of the wasp (cf. [45]); this influences the orientation of the terebra. However, the wasp also expresses steering movements of the terebra in several directions that are independent of the orientation of the metasoma (see subchapter ‘Mechanisms of terebra bending and rotation’ below).

**Penetration of the targeted host's skin**: Once the wasp has succeeded in reaching its host, it pushes its terebra straight down to its fullest extent and penetrates the skin of the beetle larva several times with rapid stabbing movements of the terebra (Additional file 1, min. 0:21–0:27, 2:33–2:47; cf. [47, 55]) achieved by fast alternate movements of the 1st valvulae.

**Injection of venom**: The host larva is usually pierced several times (cf. [45, 47]), with the 1st valvulae performing fast alternate movements. Venom is injected into the host’s body and permanently paralyses the host (Additional file 1, min. 0:21–0:27, 2:33–2:47; cf. [47]) thereby preventing its further development. This is crucial for ectoparasitoids, since movements of the host larva within a small cavity might damage the externally attached parasitoid [65].

**Assessment of the host**: The permanent paralysis of the host larva presumably allows an easier and more accurate assessment to be carried out by the female wasp, which can now actively steer its terebra (Fig. 2e–l; Additional file 1, min. 0:30–1:10, 2:48–3:17; cf. [47]; see subchapter ‘Mechanisms of terebra bending and rotation’ below). However, some passive bending of the terebra might also occur because of its deflection on the host surface. A small actively actuated bending of the apex of the terebra would therefore be sufficient to indicate the direction of the bending movement. The assessment of the host is not primarily carried out by the terebra tapping of the host surface, but by the puncture and the assessment of the host’s haemolymph (cf. [66, 67]).

**Formation of a feeding tube for host feeding**: In most parasitization attempts, the female wasps create a feeding tube. Thereby, a secretion, which is produced by the large colleterial glands [68], oozes from the entire terebra [66, 69]. The terebra is moved up and down and is also putatively rotated to a certain degree to ensure an even distribution of the secretion, which hardens in the air and remains for a couple of minutes, forming a feeding tube (cf. [47]). As a result of capillary forces, the haemolymph of the host flows upwards within the tube. The wasp now appears to lick the end of the feeding tube. The absorbed haemolymph serves both as protein-rich nutrition that is needed for egg maturation [70] and allows an assessment of the quality of the potential host [66, 67].

**Ovicide/larvacide of the competitors' eggs/larvae**: In our artificial setup, we have not tested whether the female wasps attempt to kill their conspecifics’ eggs or larvae. Ovicidal and larvicidal behaviour has not as yet been observed in *L. distinguendus*.

**Search for a suitable place for oviposition**: If the female wasp deems the host larva to be of good quality, it searches for a suitable oviposition site on the host surface. It appears to estimate the available space within the cavity to ensure that the growing larva has enough room for development (Fig. 2e–l; Additional file 1, min. 3:19–3:34; cf. [47]).

**Oviposition**: Rapid longitudinal alternate movements of the paired 1st valvulae serve to pass the egg along the terebra (Fig. 2m–p; Additional file 1, min. 1:56–2:26, 3:36–4:00; cf. [47, 71]). The diameter of the egg is significantly larger compared with that of the egg canal. The egg is thus strongly deformed during ovipositing. It does not emerge at the very apex of the terebra but is pushed out ventrally between the two paired 1st valvulae in a region about 100–200 µm proximal to the apex (Fig. 2p). Finally, the egg is attached to the surface of the host. In a few cases, it was also observed to be attached to the surface of the cavity near the host larva. Finally, the wasp withdraws its terebra. Female *L. distinguendus* only lay one egg per host [45, 55].

### Morphological structure of the musculoskeletal ovipositor system

The musculoskeletal ovipositor system of *L. distinguendus* consists of three pairs of valvulae, two pairs of valvifers, the female T9, three paired articulations and a set of nine paired muscles.

Because of its bilateral bauplan, all the ovipositor elements and muscles are paired apart from the distal region of the 2nd valvula and the female T9. Paired morphological structures are only described in the singular form in the following, i.e. the elements of the left side only, although they have a mirror image on the right side.

The anatomy of the venom system and of the female internal reproductive system is not discussed thoroughly in the following (for chalcidoids, see [19–25, 35–38, 72–79]; for parasitoid hymenopterans in general, see [26, 27]).

#### Cuticular elements of the ovipositor

**Fig. 3:**
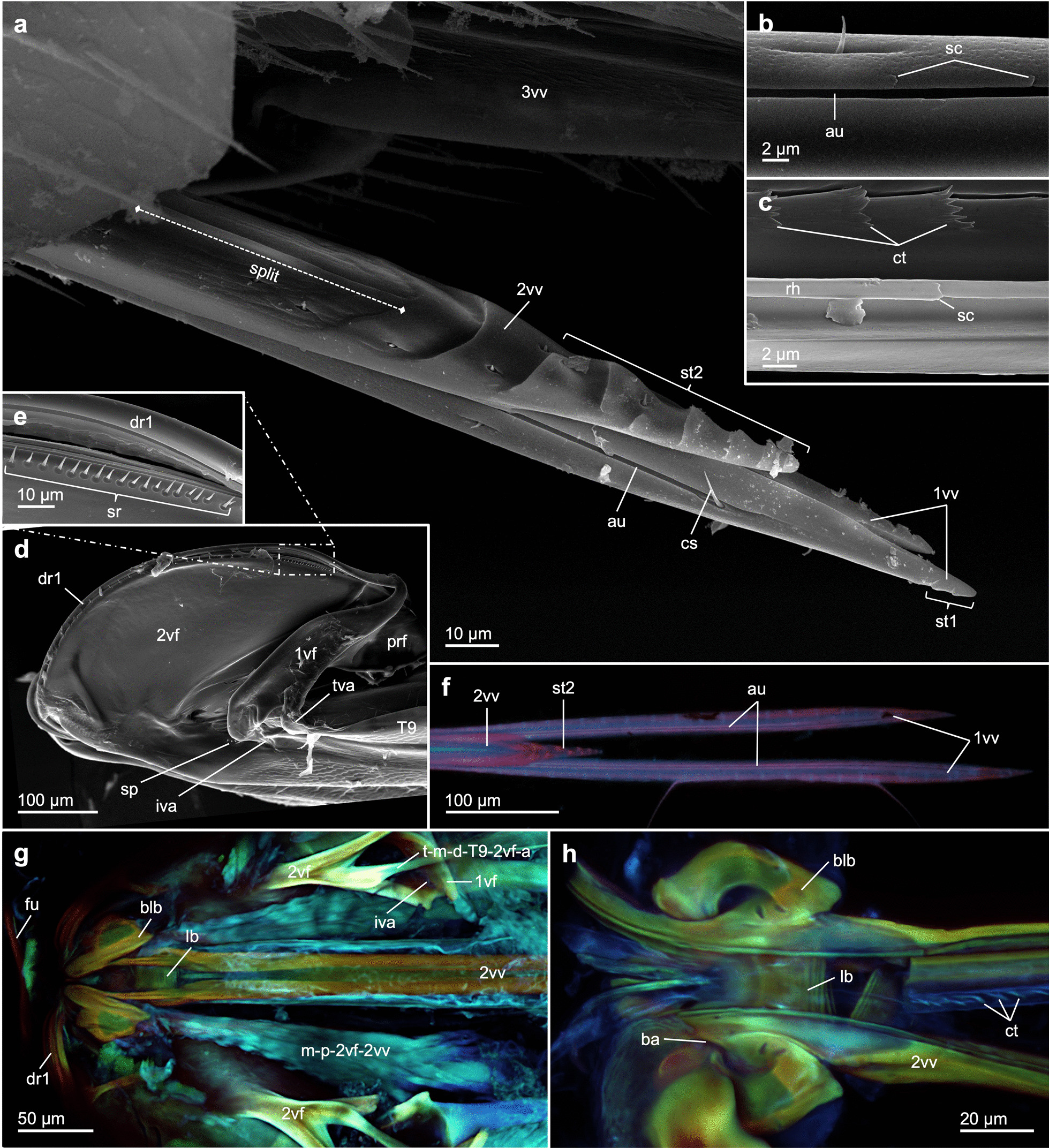
Ovipositor of *Lariophagus distinguendus*. **a**–**e** SEM images of the various ovipositor elements (left is anterior). **a** Apex of the terebra comprising the 2nd valvula and the paired 1st valvulae (dorsolateral view). The 2nd valvula is longitudinally split but fused at the apex, featuring seven sawteeth. The 1st valvula features two small apical sawteeth. Its aulax terminates pre-apically and bears a coeloconic sensillum at its apical end (for cross section cf. Fig. 4i, j). Both the 1st and 2nd valvulae bear various types of sensilla. **b** Upon removal of the 2nd valvula, the aulaces of the inner surface of the 1st valvula become visible (dorsal view), featuring distally directed scale-like structures. **c** Upon removal of the 1st valvula, the rhachis at the ventral side of the 2nd valvula becomes visible (ventral view), featuring distally directed scale-like structures similar to those of the aulax. The egg canal is formed by both the 1st and 2nd valvulae and bears microsculpture consisting of distally oriented ctenidia. **d** Anterior part of the ovipositor (lateral view). The 1st valvifer is continuous with the dorsal ramus of the 1st valvula. It is connected with the 2nd valvifer and the female T9 via the intervalvifer and tergo-valvifer articulation, respectively. The 2nd valvifer possesses a post-ramus flap and two clusters of sensilla: the sensillar patch located anteriorly to the intervalvifer articulation and the sensillar row along its dorsal margin (**e**). **f** WFM image of the apical part of the terebra of *L. distinguendus* (dorsal view, left is anterior; only the images of the DAPI and Cy5 wavelength filters are superimposed here). The cuticle of the aulaces and the sawteeth of the 2nd valvula are heavily sclerotized (as indicated by their red autofluorescence). **g**, **h** Superimposed CLSM images of the basal part of the ovipositor of *L. distinguendus* (dorsal view, left is anterior; cf. Additional file 2). The cuticle of the valvulae and the valvifers is sclerotized, whereas the ctenidia show a high content of resilin (as indicated by their blue autofluorescence; **h**). Abbreviations: 1vf: 1st valvifer; 1vv: 1st valvula; 2vf: 2nd valvifer; 2vv: 2nd valvula; 3vv: 3rd valvula; au: Aulax; ba: Basal articulation; blb: Bulb; cs: Coeloconic sensillum; ct: Ctenidium; dr1: Dorsal ramus of the 1st valvula; fu: Furcula; iva: Intervalvifer articulation; lb: Laminated bridge; m-p-2vf-2vv: Posterior 2nd valvifer-2nd valvula muscle; prf: Post-ramus flap; rh: Rhachis; sc: Scale-like structure; sp: Sensillar patch of the 2nd valvifer; sr: Sensillar row of the 2nd valvifer; st1: Sawtooth of the 1st valvula; st2: Sawtooth of the 2nd valvula; t-m-d-T9-2vf-a: Tendon of the dorsal 2nd valvifer-T9 muscle part a; T9: Female T9 (9th abdominal tergum); tva: Tergo-valvifer articulation

**Fig. 4:**
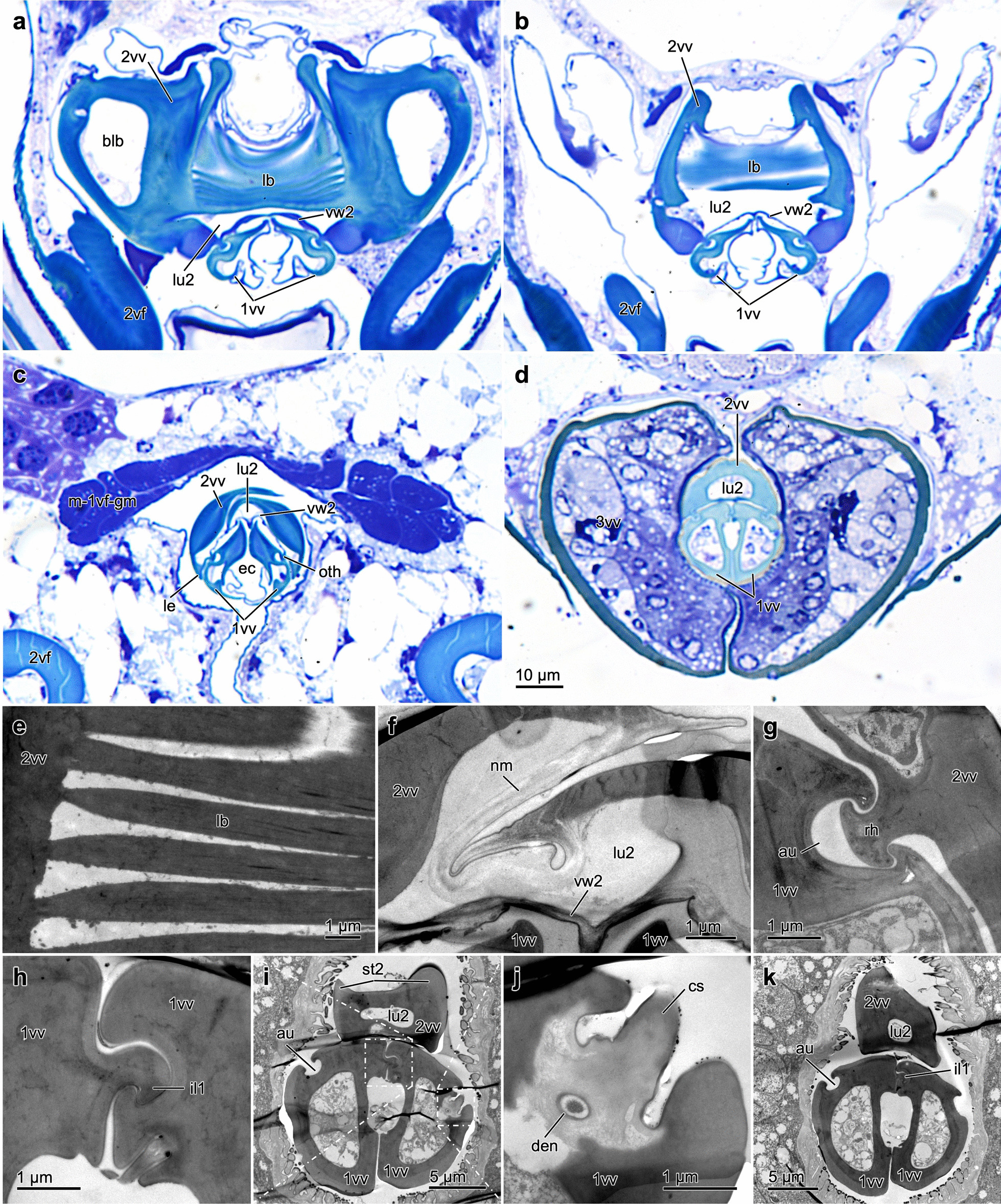
Terebra of *Lariophagus distinguendus*. **a**–**d** Light microscopical images of semithin cross sections through the terebra (from proximal to distal; scale bar in d applies to all light micrographs; positions of the sections are indicated in Fig. 6c; cf. Additional file 3). The bulbs and the laminated bridge are visible proximally. The 2nd valvula is connected with the paired 1st valvulae via the olistheter system. **e**–**k** TEM images of the terebra of *L. distinguendus*. The cuticle of the valvulae is remarkably homogenous. **e** Parts of the laminated bridge on the proximal part of the 2nd valvula (cf. a). **f** Notal membrane (cf. c). **g** Olistheter system comprising the rhachis of the 1sr valvula and the aulax of the 2nd valvula. **h**–**k** Apical part of the terebra. The olistheter-like interlock of the 1st valvulae on their dorsomedial surfaces (**h**, **I**, **k**) and the coeloconic sensillum at the apical end of one aulax are visible (**j**; for overview image, cf. Fig. 3a). Abbreviations: 1vv: 1st valvula; 2vf: 2nd valvifer; 2vv: 2nd valvula; 3vv: 3rd valvula; au: Aulax; blb: Bulb; cs: Coeloconic sensillum; den: Dendrite; ec: Egg canal; il1: Interlock of the 1st valvulae; lb: Laminated bridge; le: Lateral extensions of the 2nd valvula; lu2: Lumen of the 2nd valvula; m-1vf-gm: 1st valvifer-genital membrane muscle; nm: Notal membrane; oth: Olistheter; rh: Rhachis; st2: Sawtooth of the 2nd valvula; vw2: Ventral wall of the 2nd valvula

**Fig. 5:**
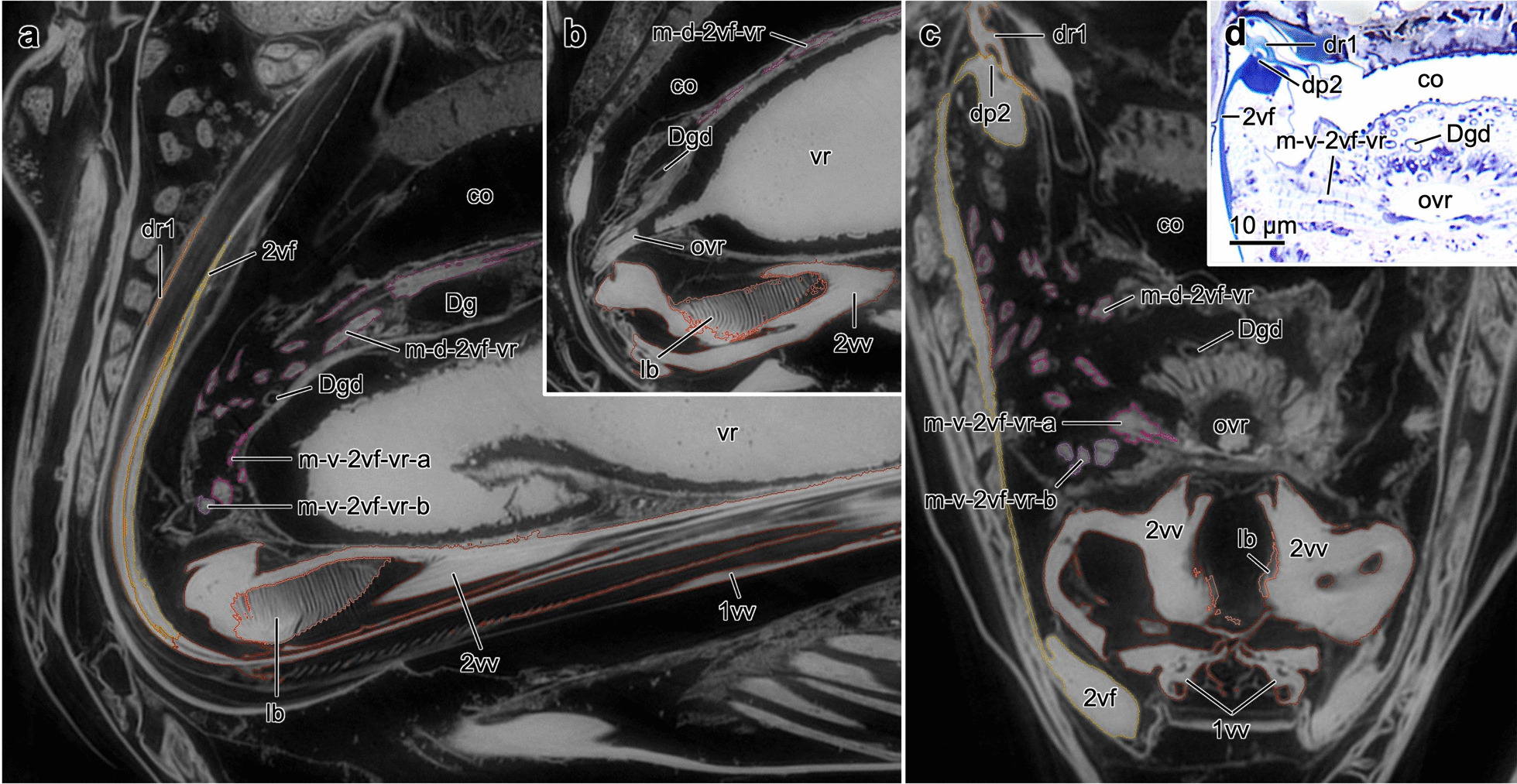
Ovipositor muscles supporting the venom and reproductive system of *Lariophagus distinguendus*. **a–c** SR-µCT images of a virtual slices through the anterior part of the ovipositor (**a,b** sagittal view, left is anterior, **c** transversal view) highlighting the muscles supporting the venom and reproductive system, the venom gland reservoir and its orifice, the Dufour’s gland and its duct, and the common oviduct (the colour labels correspond to Fig. 6 and Additional file 4). **d** Light microscopical image of a semithin cross section through the ovipositor. The ventral 2nd valvifer-venom gland reservoir muscle with its Z lines is clearly visible. Abbreviations: 1vv: 1st valvula; 2vf: 2nd valvifer; 2vv: 2nd valvula; co: Common oviduct; Dg: Dufour’s gland; Dgd: Dufour’s gland duct; dp2: Dorsal projection of the 2nd valvifer; dr1: Dorsal ramus of the 1st valvula; lb: Laminated bridge; m-d-2vf-vr: Dorsal 2nd valvifer-venom gland reservoir muscle; m-v-2vf-vr: Ventral 2nd valvifer-venom gland reservoir muscle; m-v-2vf-vr-a: Ventral 2nd valvifer-venom gland reservoir muscle part a; m-v-2vf-vr-b: Ventral 2nd valvifer-venom gland reservoir muscle part b; ovr: orifice of the venom gland reservoir; vr: Venom gland reservoir of the 2nd valvifer

**Fig. 6:**
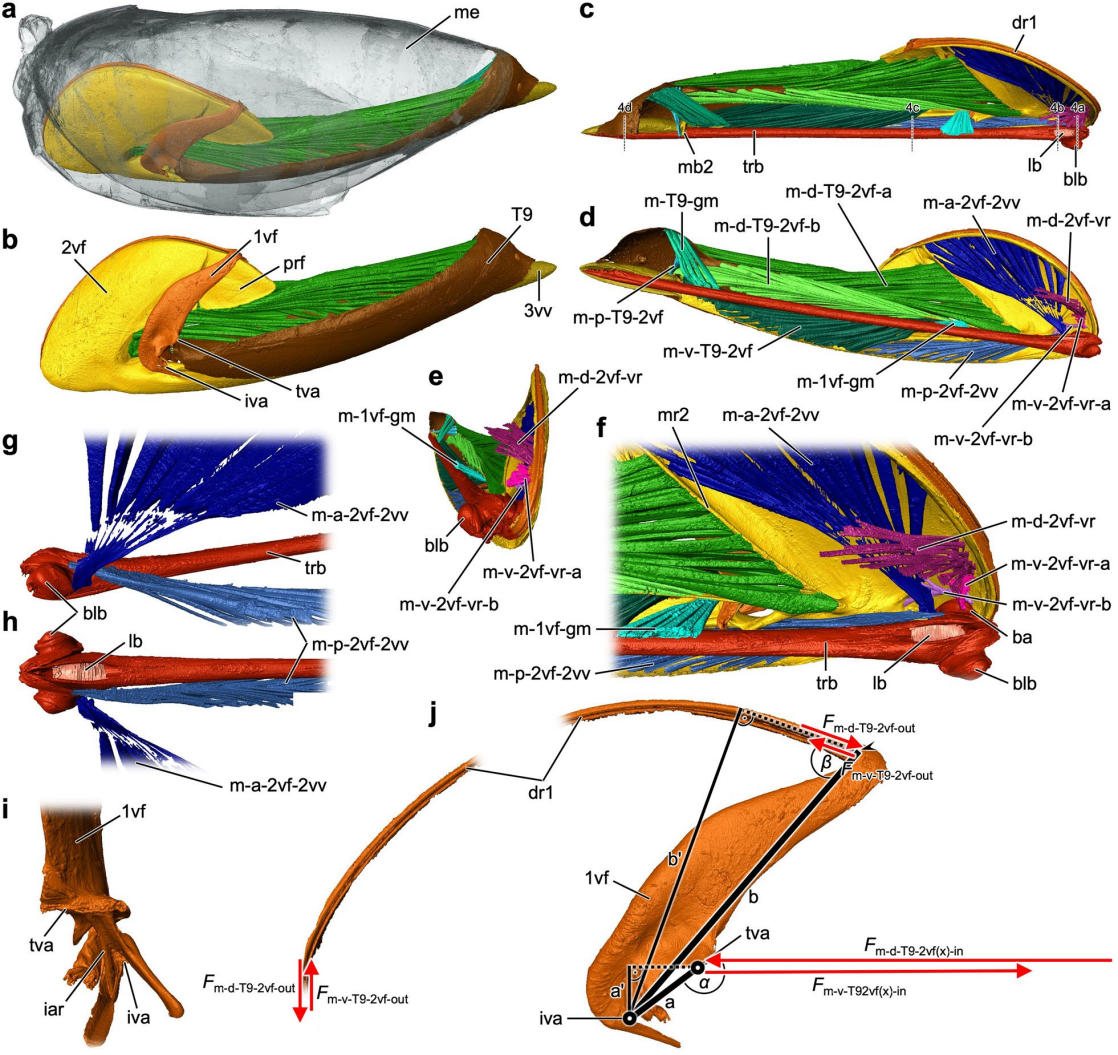
Musculoskeletal ovipositor system of *Lariophagus distinguendus*. Segmented 3D model based on SR–µCT data (perspective view; only the left side of the paired ovipositor elements are depicted; cf. Additional file 4). **a** Orientation of the ovipositor within the metasoma (lateral view, left is anterior; the metasoma is semi-transparent). **b–e** Cuticular elements, articulations and muscles of the ovipositor (**b** lateral view, left is anterior; **c** dorsal view, left is posterior, positions of sections in Fig. 4a–d are indicated here; **d** medial view, left is posterior; **e** frontal view). The ovipositor consists of the following cuticular structures (**b**): 1st valvifer, 1st valvula, 2nd valvifer, 2nd valvula, 3rd valvula and female T9 (9th abdominal tergum). The 1st valvifer is articulated with the 2nd valvifer and the female T9 via the intervalvifer and the tergo-valvifer articulation, respectively (**b**). It is continuous with the dorsal ramus of the 1st valvula (**c**). The 1st and 2nd valvulae form the terebra (1st and 2nd valvulae are not distinguished here). The various ovipositor movements are actuated by a set of nine muscles (**d**): 1st valvifer-genital membrane muscle, dorsal 2nd valvifer-venom gland reservoir muscle, ventral 2nd valvifer-venom gland reservoir muscle part a/b, anterior 2nd valvifer-2nd valvula muscle, posterior 2nd valvifer-2nd valvula muscle, dorsal T9-2nd valvifer muscle part a/b, ventral T9-2nd valvifer muscle, posterior T9-2nd valvifer muscle and T9-genital membrane muscle. **f** Anterior part of the ovipositor (dorsomedial view, left is posterior) highlighting the basal articulation and the three muscles connected to the venom gland reservoir. **g** Base of the terebra featuring the laterally placed bulbs, the laminated bridge and the insertion sites of the anterior and posterior 2nd valvifer-2nd valvula-muscles (i.e. the processus articularis and the processus musculares, respectively) and their orientation (left is anterior; **g** lateral view, **h** dorsal view). **i** Ventral part of the 1st valvifer (posterior view) highlighting the bifurcated posteroventral corner forming one part of the intervalvifer articulation, and the horizontal ridge that is part of the tergo-valvifer articulation. **j** 1st valvifer (lateral view, left is anterior) with dorsal ramus of the 1st valvula. Acting muscle forces are visualized by solid red arrows. Under the simplified assumption that the 2nd valvifer, which acts as the frame of reference, and the female T9 are guided and cannot twist but only slide towards or against each other along the anterior–posterior axis, the input force vectors *F*_m-d-T9-2vf(x)-in_ and *F*_m-v-T9-2vf(x)-in_ act in the same plane only at the tergo-valvifer articulation. The distance between the tergo-valvifer articulation (where the force is applied) and the intervalvifer articulation (pivot point/joint axis) is the anatomical inlever a, the effective (= mechanical) inlever is a'; for torques, see equations (eqs.) 1, 2. The 1st valvifer acts as a lever with the anatomical outlever b being the distance between the intervalvifer articulation and the point at which the 1st valvifer continues as dorsal ramus of the 1st valvula, the effective outlever is b', resulting in pro- or retraction forces at the dorsal ramus of the 1st valvula *F*_m-d-T9-2vf-out_ and *F*_m-v-T9-2vf-out_; see eqs. 3, 4. Abbreviations: 1vf: 1st valvifer; 1vv: 1st valvula; 2vf: 2nd valvifer; 2vv: 2nd valvula; 3vv: 3rd valvula; ba: Basal articulation; blb: Bulb; dr1: Dorsal ramus of the 1st valvula; *F*: Force; *F*_(x)_: Horizontal vector component of a force; iar: Interarticular ridge of the 1st valvifer; iva: Intervalvifer articulation; lb: Laminated bridge; m-1vf-gm: 1st valvifer-genital membrane muscle; m-a-2vf-2vv: Anterior 2nd valvifer-2nd valvula muscle; m-d-2vf-vr: Dorsal 2nd valvifer-venom gland reservoir muscle; m-d-T9-2vf-a: Dorsal T9-2nd valvifer muscle part a; m-d-T9-2vf-b: Dorsal T9-2nd valvifer muscle part b; m-p-2vf-2vv: Posterior 2nd valvifer-2nd valvula muscle; m-p-T9-2vf: Posterior T9-2nd valvifer muscle; m-T9-gm: T9-genital membrane muscle; m-v-2vf-vr-a: Ventral 2nd valvifer-venom gland reservoir muscle part a; m-v-2vf-vr-b: Ventral 2nd valvifer-venom gland reservoir muscle part b; m-v-T9-2vf: Ventral T9-2nd valvifer muscle; mb2: Median bridge of the 2nd valvifers; me: Metasoma; mr2: Medial ridge of the 2nd valvifer; prf: Post-ramus flap; T9: Female T9 (9th abdominal tergum): tva: Tergo-valvifer articulation; trb: Terebra

**1st valvula** (1vv; Figs. 2m–p, 3a, b, f, 4a–d, g–k, and 5a, c): Basally, the thin 1st valvula is continuous with the 1st valvifer via its dorsal ramus (dr1; Figs. 3d, e, g, 5a, c, d, and 6c, j). The 1st valvula has a crescent-shaped cross-section over most of its length (1vv; Fig. 4c). The aulax (au; Figs. 3a and 4g, i, k) of *L. distinguendus* does not reach the apex of the 1st valvula but tapers off around 50 µm before it. The distal end of the aulax features a coeloconic sensillum (cs; Figs. 3a and 4i, j; sensu [80]), presumably monitoring the position of the 1st valvula relative to the 2nd valvula (cf. [81]). Further sensilla can be seen at regular intervals on the lateral sides (blue ‘notches’ in Fig. 3f), which might have a mechano- and/or chemosensory function. However, the sensillar equipment of the terebra was not further investigated in this study (but see [82–84]). Dorsomedially to the aulax, the medial wall of each 1st valvula is thickened (Fig. 4c). The ventral part of the medial wall is thin and formed into a large membranous fold (when at rest) that projects inwards and overlaps ventrally (Fig. 4a–c; cf. [39]). These thin chitinous folds are considered effectively to seal the crack between the paired 1st valvulae in order to prevent the loss of venom and/or oviposition fluids [39]. The 1st valvula laterally bears two small sawteeth (st1; Fig. 3a) that are of decreasing size at its apex and that are most probably used to penetrate the substrate and the host’s skin. On the dorsomedial side of their apices, the 1st valvulae are connected by the olistheter-like interlock of the 1st valvulae (il1; Fig. 4h, i, k), presumably preventing them from being torn apart during the initial puncturing of the substrate and during drilling. The egg exits the egg canal proximad to these structures and ventrally between the paired 1st valvulae (Fig. 2p; Additional file 1, min. 1:56–2:26, 3:36–4:00). Such interlocking structures are also found in other pteromalids and some species of Aphelinidae, Chalcididae, Eulophidae, Eurytomidae, Ormyridae, Tanaostigmatidae and Trichogrammatidae [39]. In all chalcidoids, the ventral ramus of the 1st valvula is completely reduced [29] and the valvilli inside the egg canal are absent [85].

**2nd valvula** (2vv; Figs. 2m–p, 3a, c, f–h, 4a–g, i, k, and 5a–c): Proximally, the bulbs of the 2nd valvula (blb; Figs. 3g, h, 4a, and 6e–h) are basally articulated with the 2nd valvifer via the basal articulation (ba; Figs. 3h and 6f). At its basal part, the 2nd valvula bears the processus articuaris laterally on the bulbs, and the processus musculares dorsally on the anteriorly directed horn-like processes of the bulbs. On its ventral side, the 2nd valvula bears the rhachises (rh; Figs. 3c and 4g). The 2nd valvula of *L. distinguendus* consists of two longitudinally split, asymmetrically overlapping and more-heavily sclerotized halves (2vv; Figs. 3a, f–h and 4a–c; Additional file 2) that are thickened medially (2vv; Fig. 4c). The two halves are dorsally connected for most of their length by a conjunctiva called the notal membrane (nm; Fig. 4f) [17, 19–25, 40] but are fused at the apex (2vv; Figs. 3a and 4d, i, k). Proximally, the notal membrane is modified into a transversely striate band called the laminated bridge (lb; Figs. 3g, h, 4a, b, e, 5a–c, and 6c, f, h) [19, 20, 22, 40]. The modified 2nd valvula with its longitudinally split and overlapping halves presumably permit a greater distortion of the valvula and appear to be a synapomorphy for all Chalcidoidea, except for Mymaridae [39]. The ventral side is formed by the ventral wall of the 2nd valvula (vw2; Fig. 4a–c, f; sensu [86]), which extends from the base almost to the apex. This creates a lumen (lu2; Fig. 4a–d, f, i, k). The rhachises are attached to this lamella-like process over most of their length, except for the apex. Ventrolaterally to the rhachises lie lateral extensions of the 2nd valvula (le; Fig. 4c; sensu [86]). The apex of the 2nd valvulae of *L. distinguendus* features seven sawteeth that are placed laterally and staggered relative to one another (st2; Figs. 3a, f and 4i) with sensilla being found in between them. The laterally positioned sawteeth are postulated to act like a screw during the alternate rotational movements of the terebra during substrate penetration [22] and seem to be present in all chalcidoid species that undertake drilling actions [25] (Additional file 3).

**Terebra** (trb; Figs. 1, 2d–p, 3a and 6c–h): The acicular terebra consists of the paired 1st valvulae and the 2nd valvula and has a smooth surface. The terebra of *L. distinguendus* (and other chalcidoid wasps) features a single opening at the basal end, where the common oviduct (co; Fig. 5) seamlessly merges with the base of the egg canal (cf. [19–23, 25]). In chalcidoid wasps (such as *L. distinguendus* and *Microterys flavus* (Howard, 1881) (Encyrtidae) (data not yet published)), both the orifice of the venom gland reservoir (ovr; Fig. 5b–d; Additional file 4, min. 0:30–0:31) and the dorsolaterally situated Dufour’s gland duct (Dgd; Fig. 5) empty into the common oviduct (cf. [73, 77]) before the latter fuses with the egg canal (unlike in ichneumonoid wasps; cf. [16, 87]). The junction lies directly anterior to the basal articulation (ba; Figs. 3h and 6f) and is indicated by the furcula (Fig. 3g; Additional file 2, min. 0:21–0:36). The complete length of the egg canal thus functions as a conduit not only for the egg itself, but also for the expulsion of venom or other fluids during oviposition. The diameter of the terebra is even along its length (Fig. 4c; Additional file 3) between the broad basal bulbs (Figs. 3g, h and 4a,b) and the distally tapering apex (Figs. 3a and 4d, i, k). The rhachises (rh; Figs. 3c and 4g) on the ventral side of the 2nd valvula are interlocked with the aulaces (au; Figs. 3a, b and 4g, i, k) on the dorsal side of the opposing 1st valvulae via the olistheter system (oth; Fig. 4c); this enables the 1st valvulae to move along the 2nd valvulae while they are still connected to each other. The olistheters of *L. distinguendus* does not extend along the entire length of the terebra but end around 50 µm before its apex (Fig. 3a). The distally directed scale-like structures on the contact surfaces of both the rhachises and the aulaces (sc; Fig. 3b, c) presumably reduce frictional forces by minimizing the contact area of the olistheter elements [88]. However, these scale-likes structures potentially also forward a liquid lubricant from the colleterial glands (= accessory glands) to the apex of the olistheter system further to reduce friction in between the moving valvulae (cf. [89]). This arrangement might also enable particles to be continuously flushed out the olistheter system during drilling or venom injection. The scale-like structures might additionally create anisotropic conditions in the olistheter and thus prevent the 1st valvulae from randomly sliding back during drilling and piercing (cf. [31]). The longitudinally split and asymmetrically overlapping halves of the 2nd valvula presumably allow lateral sliding to occur towards or away from each other. Moreover, the rachises of *L. distinguendus* are suspended from lamellar structures of the ventral wall of the 2nd valvula (vw2; Fig. 4b, c) over their entire length, except for the apex (Fig. 4d). Thus, both the 1st and 2nd valvulae, which are connected via the olistheter system, are presumably movable in their position and may diverge tangentially. Moreover, the dorsally thickened walls of the 1st valvulae can be bent away from the midline and, in doing so, can take up the ventral membranous slack, further increasing the volume inside. This is thought to be an adaptation in several chalcidoid taxa to facilitate deformation of the terebra and temporarily to enlargement of the egg canal (ec; Fig. 4c), which is mainly formed by the two paired 1st valvulae, in order to accommodate the passing egg [39]. The olistheter system thereby must sustain the forces exerted by the egg [62, 71]. However, the maximal diameter of the apical half of the terebra is limited by the diameter of the puncture site in the substrate during oviposition. The areas of the rhachises at the basal bulbous part of the 2nd valvula presumably are also flexible (purple areas of the cuticle in Fig. 4a, b presumably indicating a higher resilin content). The internal microsculpture of the medial wall of the egg canal consists of distally orientated leaf-like ctenidia (ct; Fig. 3c, h) that contain large amounts of resilin (ct; Fig. 3h; Additional file 2, min. 0:05–0:20) and are found from the proximal basis to the region before the apex. The ctenidia help to push the deformable egg along the egg canal by alternate movements of the 1st valvulae, prevent regression [71, 88] and are also hypothesized to forward a liquid lubricant for the moving valvulae and thus to reduce friction [88, 90] and/or to produce a feeding tube. Both the 1st and 2nd valvulae have tapered apices. The terebra apex in many hymenopteran taxa is heavily sclerotized and hardened with metal atoms, such as calcium (Ca), manganese (Mn) and zinc (Zn). This enables the piercing of hard substrates, reduces wear and tear and prevents buckling [15, 62, 81, 91–94].

**3rd valvula** (3vv; Figs. 3a, 4d and 6a–d): The relatively short semi-tubular 3rd valvula of *L. distinguendus* emerges at the posterior end of the 2nd valvifer (Fig. 6a–d) and ensheaths and protects the distal part of the terebra when at rest (Fig. 4d). The distally directed microsetae on the medial surface of the 3rd valvula (Fig. 3a) are thought to be involved in the cleaning of the terebra between oviposition episodes [16, 83]. The 3rd valvula might also have a sensory function [27].

**1st valvifer** (1vf; Figs. 3d and 6a–d, i, j): The 1st valvifer of *L. distinguendus* and other chalcidoids is bow-shaped [17, 19–25, 35–37]. The anteroventral angle of the 1st valvifer features a horizontal ridge, which has a medial–lateral orientation (Fig. 6i) and which is part of the tergo-valvifer articulation (tva; Figs. 3d and 6b, i, j). The posteroventral corner of the 1st valvifer is bifurcated (Fig. 6i) and is part of the intervalvifer articulation (iva; Figs. 3d, g and 6b, i, j). The interarticular ridge (iar; Fig. 6i) lies between the two articulations and might serve mechanically to stabilize the 1st valvifer. The anterodorsal angle of the 1st valvifer is continuous with the dorsal ramus of the 1st valvula (dr1; Figs. 3d, e, g, 5a, c, d, and 6c, j), which is interlocked with the dorsal projection of the 2nd valvifer (dp2; Fig. 5c, d; cf. [31]) by a system analogous to the olistheter. This tight interlocking guides the dorsal ramus and prevents it from buckling when pushing forces are applied during the protraction of the 1st valvula. Since the dorsal ramus constantly slides around the proximal bulbous end of the 2nd valvula during pro- and retraction, the ramus needs to be flexible in the sagittal plane and thus presumably contains high proportions of the elastic rubber-like protein resilin in its cuticle (cf. [95–98]).

**2nd valvifer** (2vf; Figs. 3d, 4a–c, 5a, c, d and 6a–d, f): The 2nd valvifer is elongated and its posterior part is placed medially of the female T9 (Fig. 6b). A conjunctive, called the genital membrane (not shown), connects the ventral margins of the paired 2nd valvifers arching above the 2nd valvula. The anterior part of the 2nd valvifer of *L. distinguendus* extends dorsally in a semi-circular shape and dorsally bears the dorsal projection of the 2nd valvifer (dp2; Fig. 5c, d), which is interlocked with the dorsal ramus of the 1st valvula via an interlocking system similar to the olistheter (cf. [31]). At its posterodorsal end and posterior to its medial ridge (mr2; Fig. 6f), the anterior part of the 2nd valvifer features the post-ramus flap (prf; Figs. 3d and 6b; sensu [22]), on which the dorsal projection continues, thus allowing a greater arc of movement of the 1st valvifer and therefore a greater retraction of the 1st valvula. The 2nd valvifer features two sensillar patches: (1) the sensillar patch (sp; Fig. 3d) located anteroventrally to the intervalvifer articulation (iva; Figs. 3d, g and 6b, i, j) and (2) the row of sensilla (sr; Fig. 3e) on the dorsal margin of the 2nd valvifer. These two sensillar patches are in contact with the ventromedial side of the 1st valvifer and the dorsal ramus of the 1st valvula, respectively, and probably monitor the movements of the 1st valvula indirectly. The dorsal margins and the dorsal flanges are strengthened by cuticular ridges that putatively have a stabilizing function and prevent deformation (i.a. at the intervalvifer articulation). The posterodorsal ends of the 2nd valvifers are connected by the median bridge (mb2; Fig. 6c). The venom gland reservoir (vr; Fig. 5a, b; Additional file 2, min. 0:37–0:52; Additional file 4, min. 0:24–0:31; = acid gland reservoir sensu [19–25, 73]) is situated in between the 2nd valvifers with its proximal end lying near the base of the terebra. The Dufour’s gland (Dg; Fig. 5a; Additional file 4, min. 0:21–0:31; = alkaline gland sensu [19–25, 73]) is situated dorsolaterally to the venom gland reservoir (cf. [77, 79]).

**Female T9** (T9; Figs. 3d and 6a–d): The female T9 of *L. distinguendus* is U-shaped and situated lateral to the posterior part of the 2nd valvifers (Fig. 6b). Its elongated anteriorly projecting arms articulate with the 1st valvifers via the tergo-valvifer articulations (tva; Figs. 3d and 6b, i, j). The cordate apodeme (not shown) on the anterior margin of the female T9 is located posterior to the articulation. The dorsal margins are strengthened by the anterior flange of T9, which presumably mechanically stabilizes the female T9 during oviposition. Medially, the anterior flange of T9 bifurcates and forms a dorsomedial crest-like ridge that runs almost the entire length of the female T9. This ridge serves as a muscle attachment area both medially and laterally and presumably increases the mechanical stability of the female T9.

#### Articulations of the musculoskeletal ovipositor system

**Basal articulation** (ba; Figs. 3h and 6f): The two articular surfaces of this ball-and-socket-like articulation are located on the socket-like pars articularis of the anteroventral part of the 2nd valvifer and the ball-like processus articulated laterally on the bulb of the 2nd valvula. This rotational joint presumably also allows some pivotal and rotational movements of the 2nd valvula and thus of the whole terebra.

**Intervalvifer articulation** (iva; Figs. 3d, g and 6b, i, j): The 1st and 2nd valvifer are connected via the intervalvifer articulation, a rotational joint that allows a rotation of the 1st valvifer in the sagittal plane only [32]. This articulation consists of the bifurcated posteroventral corner of the 1st valvifer (iva; Fig. 6i), which encloses the articulation site at the 2nd valvifer. Thereby, one furcal structure of the 1st valvifer is placed medially and one laterally to the 2nd valvifer.

**Tergo-valvifer articulation** (tva; Figs. 3d and 6b, i, j): The 1st valvifer lies adjacent to the female T9 via the tergo-valvifer articulation, which is situated dorsally to the intervalvifer articulation. It is a rotational joint that allows the 1st valvifer to rotate in the sagittal plane only [32]. This articulation consists of a horizontal ridge at the 1st valvifer (tva; Fig. 6i) and a corresponding counterpart at the female T9 situated near the cordate apodeme.

#### Ovipositor muscles

In total, nine paired ovipositor muscles have been identified that drive and actuate the associated skeletal apparatus (Table 1). Three of these muscles (i.e. the 1st valvifer-genital membrane muscle, the ventral 2nd valvifer-venom gland reservoir muscle and the T9-genital membrane muscle) are described here for the first time in chalcidoids.

**Table 1:** Ovipositor muscles of *Lariophagus distinguendus* (abbreviations in brackets) and their origin, insertion (cf. Additional file 4) and presumed function as verified in the present contribution

Muscle name (abbreviation)	Origin	Insertion	Presumed functions
1st valvifer-genital membrane muscle (m-1vf-gm)*	Medial surface of the posteroventral part of the 1st valvifer, at the centre between the tergo-valvifer and the intervalvifer articulation (Fig. 6c, d, f)	Anteriorly at the genital membrane (Fig. 4c)	Tensor muscle for stabilization of the 1st valvifer during ovipositor movements
Dorsal 2nd valvifer-venom gland reservoir muscle (m-d-2vf-vr)	Medial surface of the most anterior part of the 2nd valvifer (Fig. 6c–f)	Dorsally at the anterior part of the venom gland reservoir (Fig. 5a, b)	Supporting the discharge of venom gland reservoir secretion and probably of Dufour’s gland secretion, tensor muscle for stabilization of the 2nd valvifer during ovipositor movements
Ventral 2nd valvifer-venom gland reservoir muscle part a (m-v-2vf-vr-a)*	Medial surface of the most anterior part of the 2nd valvifer, ventrally to the origin of m-d-2vf-vr (Fig. 6d–f)	Laterally at the orifice of the venom gland reservoir (Fig. 5c, d)	Increasing the diameter of the orifice of the venom gland reservoir, thus controlling the venom discharge
Ventral 2nd valvifer-venom gland reservoir muscle part b (m-v-2vf-vr-b)*	Medial surface of the most anterior part of the 2nd valvifer, posteroventrally to the origin of m-v-2vf-vr-a (Fig. 6d–f)	Laterally at the orifice of the venom gland reservoir, ventrally to the insertion of m-v-2vf-vr-a, shortly before the orifice of the venom gland reservoir enters the common oviduct (Fig. 5c, d)	
Anterior 2nd valvifer-2nd valvula muscle (m-a-2vf-2vv)	Medial region along the anterodorsal arch of the 2nd valvifer (Fig. 6c, d)	At the processus articularis on the 2nd valvula, laterally at the bulbs of the 2nd valvula (Fig. 6f–h)	Pulling of the corresponding bulb of the 2nd valvula dorsad (thus inducing lateral bending movements of the terebra) in the active probing position, assistance in the rotation of the terebra during oviposition process, elevator of the terebra back into its resting position (once withdrawn from the substrate), holding the terebra in resting position
Posterior 2nd valvifer-2nd valvula muscle (m-p-2vf-2vv)	Medial region along the ventral part of the 2nd valvifer (Fig. 6c, d)	At the processus musculares on the 2nd valvula, dorsally at the anteriorly directed horn-like processes of the bulbs of the 2nd valvula (Fig. 6f–h)	Rotation of the terebra during oviposition process, inducing partial deformation of the 2nd valvula by moving its two halves tangentially towards each other, holding of the terebra in the active probing position
Dorsal T9-2nd valvifer muscle part a (m-d-T9-2vf-a)	Lateral region along the posterodorsal part of the female T9, laterally along its dorsomedial ridge (Fig. 6a–d)	Anterior section of the dorsal flange of the 2nd valvifer, posterior to its medial ridge (Fig. 6c, d, f)	Protractor of the 1st valvula: moves the 2nd posteriorly and the female T9 anteriorly towards each other, causing the 1st valvifer to tilt anteriorly and thus the 1st valvula to slide distally relative to the 2nd valvula
Dorsal T9-2nd valvifer muscle part b (m-d-T9-2vf-b)	Medial region along the posterodorsal part of the female T9, ventromedially to its dorsomedial ridge (Fig. 6c, d)	Anterior section of the dorsal flange of the 2nd valvifer via a tendon, ventrally to the insertion of m-d-T9-2vf-a (Fig. 6c, d)	
Ventral T9-2nd valvifer muscle (m-v-T9-2vf)	At the cordate apodeme at the anterior margin of the female T9, posteriorly to the tergo-valvifer articulation (Fig. 6c, d)	Medial surface along the posterior section of the dorsal flange of the 2nd valvifer (Fig. 6c, d)	Retractor of the 1st valvula: moves the 2nd anteriorly and the female T9 posteriorly apart from each other, causing the 1st valvifer to tilt posteriorly and thus the 1st valvula to slide proximally relative to the 2nd valvula
Posterior T9-2nd valvifer muscle (m-p-T9-2vf)	Medial surface of the posterodorsal part of the female T9 (Fig. 6c, d)	Median bridge of the 2nd valvifers	Tensor muscle for stabilization by holding the posterior part of the 2nd valvifer in position during ovipositor movements
T9-genital membrane muscle (m-T9-gm)*	Medial surface of the posterodorsal part of the female T9, dorsally of the origin of m-p-T9-2vf (Fig. 6c, d)	Posteriorly at the genital membrane	Tensor muscle for stabilization

The muscles marked with * are described here for the first time in chalcidoids

**1st valvifer-genital membrane muscle** (m-1vf-gm; Figs. 4c and 6d, e, f): This muscle is the only muscle of the 1st valvifer. It originates at the medial surface of the posteroventral part of the 1st valvifer, i.e. at the centre between the tergo-valvifer and the intervalvifer articulation (Fig. 6c, d, f), and inserts anteriorly on the genital membrane (Fig. 4c). We here describe the m-1vf-gm for the first time in Chalcidoidea. Previous authors (e.g. [17, 19–25, 36]) might have overlooked its presence because of to its minute size.

**Dorsal 2nd valvifer-venom gland reservoir muscle** (m-d-2vf-vr; Figs. 5a–c and 6d, e, f): This muscle originates at the medial surface of the most anterior part of the 2nd valvifer (Fig. 6c–f) and inserts dorsally at the anterior part of the venom gland reservoir (Fig. 5a, b), which is located ventrally of the common oviduct. Most previous authors (e.g. [17, 19–25]) have overlooked the presence of this muscle; it was only mentioned by [73].

**Ventral 2nd valvifer-venom gland reservoir muscle** (m-v-2vf-vr-a/b; Figs. 5a, c, d and 6d–f): This muscle forms two distinct bundles. Its anterodorsal part (m-v-2vf-vr-a) originates at the medial surface of the most anterior part of the 2nd valvifer, ventrally to the origin region of the dorsal 2nd valvifer-venom gland reservoir muscle (Fig. 6d–f), and inserts laterally at the orifice the venom gland reservoir (Fig. 5c, d). The other part (m-v-2vf-vr-b) originates at the medial surface of the anterior part of the 2nd valvifer, posteroventrally to the origin region of part a (Fig. 6d–f), and inserts laterally at the orifice of the venom gland reservoir, ventrally to the insertion of part a and shortly before the orifice of the venom gland reservoir enters the common oviduct (Fig. 5c, d). To our knowledge, this muscle has also not yet been described in chalcidoids (but see [99–101] for the description of a similar set of muscles in ants).

**Anterior 2nd valvifer-2nd valvula muscle** (m-a-2vf-2vv; Fig. 6d, f, g, h): This muscle originates at the medial region along the anterodorsal arch of the 2nd valvifer (Fig. 6c, d) and inserts at the processus articularis, located laterally on the bulbs of the 2nd valvula (Fig. 6f–h).

**Posterior 2nd valvifer-2nd valvula muscle** (m-p-2vf-2vv; Fig. 6d, f, g, h): This muscle originates at the medial region along the ventral part of the 2nd valvifer (Fig. 6c, d) and inserts at the processus musculares, located dorsally on the anteriorly directed horn-like processes of the bulbs of the 2nd valvula (Fig. 6f–h).

**Dorsal T9-2nd valvifer muscle** (m-d-T9-2vf-a/b; Fig. 6d): This muscle is modified in its insertion and forms two distinct muscle bundles. One part of this muscle (m-d-T9-2vf-a) originates at the lateral region along the posterodorsal part of the female T9, i.e. laterally along its dorsomedial ridge (Fig. 6a–d), and inserts at the anterior section of the dorsal flange of the 2nd valvifer, posterior to its medial ridge (Fig. 6c, d, f). The other part (m-d-T9-2vf-b) originates at the medial region along the posterodorsal part of the female T9, i.e. ventromedially to its dorsomedial ridge (Fig. 6c, d), and inserts at the anterior section of the dorsal flange of the 2nd valvifer via a tendon (t-m-d-T9-2vf-a; Fig. 3g), located ventrally to the insertion region of m-d-T9-2vf-a (Fig. 6c, d).

**Ventral T9-2nd valvifer muscle** (m-v-T9-2vf; Fig. 6d): This muscle originates at the cordate apodeme, which is located at the anterior margin of the female T9, posteriorly to the tergo-valvifer articulation (Fig. 6c, d), and inserts at the medial surface along the posterior section of the dorsal flange of the 2nd valvifer (Fig. 6c, d).

**Posterior T9-2nd valvifer muscle** (m-p-T9-2vf; Fig. 6d): This muscle originates at the medial surface of the posterodorsal part of the female T9 (Fig. 6c, d) and inserts at the median bridge of the 2nd valvifers. Previous studies on the chalcidoid ovipositor [17, 19–25] report only one muscle originating in the posterior region of the female T9. The authors presumably were unable to distinguish this muscle from the T9-genital membrane muscle described below.

**T9-genital membrane muscle** (m-T9-gm; Fig. 6d): This muscle originates at the medial surface of the posterodorsal part of the female T9, dorsally of the origin region of the posterior T9-2nd valvifer muscle (Fig. 6c, d), and inserts posteriorly at the genital membrane. We here describe the m-T9-gm for the first time in Chalcidoidea.

### Mechanics and mode of function of the ovipositor system

The set of nine paired ovipositor muscles in *L. distinguendus* comprises two pairs of two antagonistically working muscles that are mainly responsible for the various ovipositor movements, three muscles stabilizing the musculoskeletal system, and two muscles related to the function of the venom gland reservoir (Table 1).

**Fig. 7:**
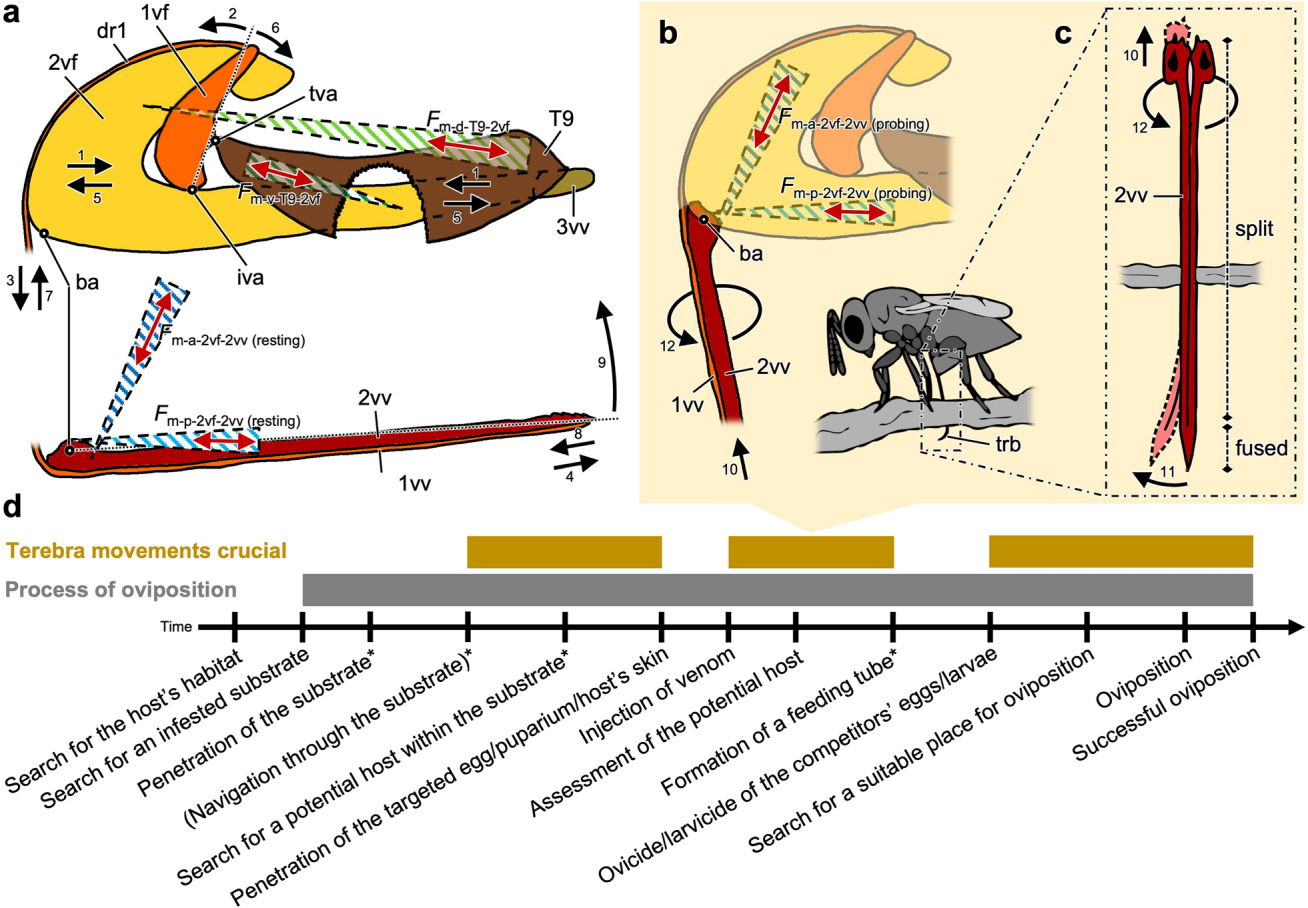
Mechanisms driving the various ovipositor movements of *Lariophagus distinguendus*, and the importance of the terebra movements during the oviposition process. **a–c** Functional model of the mechanisms driving the various ovipositor movements in the resting and the active probing position (only the left side of the paired ovipositor elements are depicted; not to scale). Acting (input) muscle forces are visualized by solid red arrows and resulting (output) movements by solid black arrows. **a** Mechanism of the tilting movement of the 1st valvifer and the resulting pro- and retraction of the 1st valvulae (lateral view, left is anterior). Only the two pairs of antagonistically working muscles that are responsible for these movements (m-a-2vf-2vv/m-p-2vf-2vv and m-d-T9-2vf/m-v-T9-2vf) are represented in simplified terms. The muscles stabilizing the ovipositor system (m-1vf-gm; m-p-T9-2vf; m-T9-gm) and those supporting the venom and reproductive systems (m-d-2vf-vr; m-v-2vf-vr) are not shown. Contraction of (both parts of) m-d-T9-2vf (*F*_m-d-T9-2vf_) slides the 2nd valvifer posteriorly and the female T9 anteriorly towards each other (arrow 1), thus indirectly causing the 1st valvifer to tilt anteriorly (arrow 2). This is possible because the 1st valvifer is articulated with both the 2nd valvifer and the female T9 via the intervalvifer and tergo-valvifer articulation, respectively. The 1st valvifer thereby functions as a lever arm that transmits the movement to the dorsal ramus of the 1st valvula (arrow 3) and consequently causes a protraction of the 1st valvula (arrow 4). M-p-T9-2vf and m-T9-gm thereby presumably stabilize the system by holding the 2nd valvifer and the female T9 in position and preventing them from rotating around the articulations. Contraction of m-v-T9-2vf (*F*_m-v-T9-2vf_) slides the 2nd valvifer anteriorly and the female T9 posteriorly apart from each other (arrow 5), thus causing the 1st valvifer to tilt posteriorly (arrow 6). This movement is transmitted via the dorsal ramus of the 1st valvula (arrow 7) and consequently causes its retraction (arrow 8). When the terebra is withdrawn from the substrate, a contraction of the m-a-2vf-2vv (*F*_m-a-2vf-2vv_) presumably causes the bulbs to pivot posteriorly around the basal articulation, thus elevating the 2nd valvula and therefore the whole terebra back into its resting position (arrow 9). **b**, **c** Mechanisms of the bending and rotational movements of the terebra (**b** lateral view, left is anterior; **c** dorsal view; schematic drawing of wasp in lateral view). During oviposition, a contraction of m-a-2vf-2vv cannot elevate the terebra back towards its resting position (as described in other hymenopteran taxa), because the terebra is anchored at the puncture site in the substrate. In this situation, a contraction of one of the paired m-a-2vf-2vv (*F*_m-a-2vf-2vv_) in the active probing position pulls the corresponding bulb and thus one half of the longitudinally split and asymmetrically overlapping 2nd valvula dorsad along its longitudinal axis (arrow 10) because of the orientation of the muscle and the resulting direction of the force vector. Since the halves of the 2nd valvula are fused at the apex, this movement causes the distal part of the terebra (i.e. the part inside the cavity in the substrate) to bend to the left or right: a contraction of the left m-a-2vf-2vv causes the 2nd valvula and thus the whole terebra to bend to the left (arrow 11), a contraction of the right m-a-2vf-2vv causes a bend to the right. In addition, a contraction of one of the m-p-2vf-2vv (*F*_m-p-2vf-2vv_) in the active probing position presumably causes the 2nd valvula and thus the whole terebra to rotate back and forth at the basal articulation along its longitudinal axis to a certain degree: a contraction of the left m-p-2vf-2vv causes the 2nd valvula and thus the whole terebra to rotate anti-clockwise when viewed from the dorsal side (arrow 12), whereas a contraction of the right m-p-2vf-2vv results in a clockwise rotation (cf. Additional file 1). Contractions of the m-a-2vf-2vv might support these rotational movements. The rotation allows the bending movements to take effect in different directions. **d** Timeline of the oviposition process of an idiobiont ectoparasitoid wasp highlighting the importance of terebra movements during the various stages (stages in parenthesis do not occur in *L. distinguendus*; stages with * do not occur in all parasitoid lifestyles but are particularly notable in idiobiont ectoparasitoids). Abbreviations: 1vf: 1st valvifer; 1vv: 1st valvula; 2vf: 2nd valvifer; 2vv: 2nd valvula; 3vv: 3rd valvula; ba: Basal articulation; dr1: Dorsal ramus of the 1st valvula; *F*: Force; iva: Intervalvifer articulation; m-a-2vf-2vv: Anterior 2nd valvifer-2nd valvula muscle; m-d-T9-2vf: Dorsal T9-2nd valvifer muscle; m-p-2vf-2vv: Posterior 2nd valvifer-2nd valvula muscle; m-v-T9-2vf: Ventral T9-2nd valvifer muscle; T9: Female T9 (9th abdominal tergum); tva: Tergo-valvifer articulation; trb: Terebra

**Depression and elevation of the terebra**: The 2nd valvula is connected with the 2nd valvifer by a rotational joint called the basal articulation (ba; Figs. 3h, 6f and 7a). Two muscles (m-a-2vf-2vv, m-p-2vf-2vv) insert at the bulbous region around this articulation. The insertion region of the posterior 2nd valvifer-2nd valvula muscle (m-p-2vf-2vv; Fig. 6f–h) at the 2nd valvula is located dorsal of the basal articulation, whereas its region of origin at the 2nd valvifer is located posteroventral to it (Fig. 6c, d). Taxa from other superfamilies use the m-p-2vf-2vv to depress their terebra towards an active probing position (e.g. Ichneumonoidea [31, 32]). However, female *L. distinguendus* have never been observed to depress their terebra in such a manner. Instead, these wasps bend their whole metasoma downwards to bring their terebra into the drilling position. Once the apex of the terebra is engaged in the substrate, the metasoma is lifted upwards again, while the terebra remains in its depressed position (Fig. 2b–d; Additional file 1, min. 0:06–0:11; cf. [45, 47]). This behaviour has also been reported for other pteromalids [40, 46] and species of Torymidae [23], Eurytomidae [21], Encyrtidae (data not yet published) and Eulophidae [19]. Therefore, in pteromalids (and possibly also in other chalcidoid taxa), we assume that the m-p-2vf-2vv is adapted in its function (see paragraph ‘Rotation of the terebra’ of the subchapter ‘Mechanisms of terebra bending and rotation’ below). During this indirect depression of the terebra, the bulbs of the 2nd valvula might be pulled out of the socket-like anterior ends of the 2nd valvifer ventrally by pushing them slightly apart, resulting in a slight translation of the pivot point (= joint axis or fulcrum) of the basal articulation (cf. [32]). The insertion region of the anterior 2nd valvifer-2nd valvula muscle (m-a-2vf-2vv; Fig. 6f–h) at the 2nd valvula is situated posteroventrally of both the basal articulation and the insertion region of m-p-2vf-2vv, whereas its origin at the 2nd valvifer is located posterodorsally of this articulation (Fig. 6c, d). After an oviposition attempt, the terebra is withdrawn from the substrate. Since slender structures such as the terebra can support much higher tensile than compressive stresses, the withdrawal does not damage it [62]. A contraction of the anterior 2nd valvifer-2nd valvula muscle (*F*_m-a-2vf-2vv_; Fig. 7a) presumably initiates the elevation of the terebra (arrow 9; Fig. 7a; Table 1). The passive rebound of the bulbs of the 2nd valvula into the socket-like anterior ends of the 2nd valvifer presumably further supports the elevation of the terebra passively and helps to stabilize it in its resting position (cf. elevation of the terebra in ceraphronoids, which completely lack the m-a-2vf-2vv [102]). The anatomical cluster comprising the 2nd valvifer, the 2nd valvula and the two muscles connecting them is a simple mechanical system in which the 2nd valvula is a two-armed class 1 lever, whereby the effective (= mechanical) inlever arm and the joint angle (attachment angle) of m-a-2vf-2vv change over the range of motion (cf. [31]).

**Pro- and retraction of the 1st valvulae**: Three muscles (m-d-T9-2vf, m-v-T9-2vf, m-p-T9-2vf) connect the 2nd valvifer with the female T9. Both of these cuticular structures are connected with the 1st valvifer via the intervalvifer articulation and the tergo-valvifer articulation (iva/tva; Figs. 3d, 6b, i, j and 7a), respectively. The insertion region of both parts of the dorsal T9-2nd valvifer muscle (m-d-T9-2vf-a/b; Fig. 6c, d) at the 2nd valvifer are situated anterodorsally, whereas their regions of origin at the female T9 are located posterodorsally of both articulations (Fig. 6c, d). A simultaneous contraction of m-d-T9-2vf-a and m-d-T9-2vf-b (summarized as *F*_m-d-T9-2vf_; Fig. 7a) slides the 2nd valvifer posteriorly with respect to the female T9 (arrow 1; Fig. 7a). This causes the 1st valvifer to tilt anteriorly (arrow 2; Fig. 7a), because it is articulated with both the 2nd valvifer and the female T9 via rotational joints. The 1st valvifer acts as a lever that transforms its tilting movement to the dorsal ramus of the 1st valvula (arrow 3; Fig. 7a). Its tight interlocking with the dorsal projection of the 2nd valvifer prevents it from buckling and transmits the movements to the apex of the 1st valvula, causing it to slide distally relative to the 2nd valvula, i.e. to protract (arrow 4; Fig. 7a; Table 1). In the active probing position, the dorsal ramus is less curved, which presumably reduces friction [32]. The region of origin of the antagonistically acting ventral T9-2nd valvifer muscle at the female T9 (m-v-T9-2vf; Fig. 6c, d) is situated posterodorsally near the intervalvifer articulation and posterior to the tergo-valvifer articulation, whereas its insertion region at the 2nd valvifer is located posteroventrally of both these articulations (Fig. 6c, d). Its contraction (*F*_m-v-T9-2vf_; Fig. 7a) slides the 2nd valvifer anteriorly with respect to the female T9 (arrow 5; Fig. 7a), thus indirectly causing the 1st valvifer to tilt posteriorly (arrow 6; Fig. 7a) and the 1st valvula to slide proximally relative to the 2nd valvula, i.e. to retract (arrows 7, 8; Fig. 7a; Table 1). The vibration-like rapid reciprocal alternate pro- and retracting movements of the 1st valvulae are crucial for drilling and precise egg laying (Fig. 2m–p; Additional file 1, min. 1:11–1:35, 1:56–2:22, 3:36–4:00; cf. [32, 44, 47]). The following assumptions have been made for a simplified estimation of the torques (*M*) exerted by the forces of the dorsal and ventral T9-2nd valvifer muscles (*F*_m-d-T9-2vf_/*F*_m-v-T9-2vf_; Fig. 7a): (1) The 2nd valvifer acts as the frame of reference; therefore, the intervalvifer articulation (iva; Figs. 6i, j and 7a) acts as a pivot point around which the 1st valvifer tilts; (2) the movements of 2nd valvifer and the female T9 are constrained by the musculoskeletal system and cannot twist around the articulations but only slide telescopically towards or against each other along the anterior–posterior axis; and (3) frictional forces in the system can be neglected. In reality, all cuticular elements can move relatively to each other. However, under these assumptions, the horizontal force vector components acting in the anterior–posterior axis (*F*_m-d-T9-2vf(x)-in_/*F*_m-v-T9-2vf(x)-in_; Fig. 6j) act at the 1st valvifer at the tergo-valvifer articulation (tva; Figs. 6i, j and 7a). Therefore, the torques (*M*) of *F*_m-d-T9-2vf_ and *F*_m-v-T9-2vf_ that act at the intervalvifer articulation in the resting position can be estimated by using the horizontal vector components (*F*_m-d-T9-2vf(x)-in_/*F*_m-v-T9-2vf(x)-in_; Fig. 6j) of the maximum force of a muscle, the length of the anatomical inlever arm (a; Fig. 6j), i.e. the distance between the intervalvifer and the tergo-valvifer articulation, and the joint angle (*α*; Fig. 6j) according to the equations:

1 $$M_{{\text{m-d-T9-2vf}}} = F_{{{\text{m-d-T9-2vf}}\left( {\text{x}} \right){\text{-in}}}} \cdot {\text{a}} \cdot \sin(\alpha)$$

2 $$M_{{\text{m-v-T9-2vf}}} = F_{{{\text{m-v-T9-2vf}}\left( {\text{x}} \right){\text{-in}}}} \cdot {\text{a}} \cdot \sin(\alpha)$$

The 1st valvifer acts as a one-armed class 3 lever (force arm smaller than load arm) with the anatomical inlever (a; Fig. 6j) and the anatomical outlever (b; Fig. 6j), the latter being the distance between the intervalvifer articulation and the point at which the 1st valvifer continues as dorsal ramus of the 1st valvula (arrowhead; Fig. 6j). The resulting pro- and retracting forces at the dorsal ramus of the 1st valvula (*F*_m-d-T9-2vf-out_/*F*_m-v-T9-2vf-out_; Fig. 6j) can be estimated using the horizontal vector components (*F*_m-d-T9-2vf(x)-in_/*F*_m-v-T9-2vf(x)-in_; Fig. 6j) of the forces acting on the 1st valvifer at the tergo-valvifer articulation, the length of the effective inlever arm (a' = a · sin(*α*); Fig. 6j) and the effective outlever arm (b' = b · sin(*β*); Fig. 6j) according to the equations:

3 $$F_{{\text{m-d-T9-2vf-out}}} = \left( {F_{{{\text{m-d-T9-2vf}}\left( {\text{x}} \right){\text{-in}}}} \cdot {\text{a'}} } \right)/ \,\, {\text{b'}}$$

4 $$F_{{\text{m-v-T9-2vf-out}}} = \left( {F_{{{\text{m-v-T9-2vf}}\left( {\text{x}} \right){\text{-in}}}} \cdot {\text{a'}} } \right)/ \, \,{\text{b'}}$$

The shape of the 1st valvifer and the positions of the intervalvifer and the tergo-valvifer articulations influence the way that the 1st valvula is moved. A comparatively high quotient of the effective outlever to the effective inlever (b'/a' ratio), as observed in *L. distinguendus* (and other chalcidoid taxa [17, 21–24]), results in a smaller force output but an increase in the potential maximum velocity and mechanical deflection, i.e. an increase in the speed and the movement distance of the 1st valvula [31, 32, 87, 102].

**Stabilization of the ovipositor**: The small 1st valvifer genital membrane muscle (m-1vf-gm; Figs. 4c and 6d–f) presumably acts as a tensor muscle and stabilizes the 1st valvifers when performing the rapid pivoting movements during substrate drilling, host envenomation and oviposition (Table 1). Additionally, it might also contribute to bringing the 1st valvula into its aligned configuration [32]. The tension of both the T9-genital membrane muscle (m-T9-gm; Fig. 6d) and the posterior T9-2nd valvifer muscle (m-p-T9-2vfv; Fig. 6d) might predominantly serve the stabilization of the ovipositor during oviposition by holding the 2nd valvifers and the female T9 in position and preventing them from rotating around the articulations (Table 1). M-p-T9-2vf is also hypothesized to provide the 3rd valvulae with a certain degree of mobility [20, 22]. However, given its insertion on the median bridge of the 2nd valvifer, this is only possible if a contraction of this muscle is able to cause an elastic deformation of the median bridge, which is connected with the base of the 3rd valvula.

**Support of the venom and reproductive system**: The dorsal 2nd valvifer-venom gland reservoir muscle (m-d-2vf-vr; Figs. 5a–c and 6d–f) inserts dorsally at the venom gland reservoir. Its contraction presumably supports the discharge of the secretion from both the venom gland reservoir and the Dufour’s gland (Table 1; cf. [73, 99, 100, 103]). However, given its medial insertion, it might also act as a tensor muscle stabilizing the 2nd valvifer during the ovipositor movements. The two parts of the ventral 2nd valvifer-venom gland reservoir muscle (m-v-2vf-vr-a/b; Figs. 5a, c, d and 6d–f) insert laterally at the orifice the venom gland reservoir shortly before the latter enters the common oviduct. A contraction of this muscle presumably increases the diameter of the orifice, thereby controlling the venom discharge (Table 1). [35] described a muscle originating at the medial walls of the abdominal sternum 7 and inserting at the vagina; this muscle is postulated to assist in the expulsion of eggs.

### Mechanisms of terebra bending and rotation

Various joint-free movement mechanisms have been described in animals (reviewed in [104]), and a variety of steering mechanisms, summarized in the following, have been proposed for the terebra of parasitoid wasps alone (cf. [62]).

The passive bending of the terebra originates from mechanical interactions of the inserted terebra with the surrounding substrate, e.g. the movements of the terebra of the fruit-fly parasitoid *Diachasmimorpha longicaudata* (Ashmead, 1905) (Braconidae) originate from the interplay between the surrounding substrate and relative movements of the valvulae. The relative position of the individual valvulae featuring geometrically asymmetric bevelled apices create various degrees of geometric asymmetry of the terebra apex. Consequently, the asymmetric substrate reaction forces acting on the apex push it away from a straight path [44], leading to a passive bending of the terebra, which is further facilitated by stiffness gradients in the cuticle of the apical part of the valvulae [105]. The structure and spacing of the ovipositor teeth are also thought to be involved in the passive bending movements of the terebra within plant substrates [106]. Passive bending mechanisms of the terebra are also likely to occur in species of Cynipidae (‘ovipositor searching’ sensu [107]) and Figitidae [108, 109] while they search for potential host larvae that live in plant substrates, and in species of Torymidae [110] and Agaonidae (fig wasps) [81, 106, 111] during the navigation of the terebra through the plant substrate.

The active bending of the terebra occurs when the bending moments originate from the relative movements of the valvulae, actuated by muscles inside the metasoma, e.g. (1) in species of the Aulacidae and Gasteruptiidae, abrupt terminal stops of the aulaces or protuberances in the ventrolateral side of the 2nd valvula interact with the rhachises or corresponding bosses of the 1st valvulae when the 1st valvulae are protracted and, thus, allow some dorsal bending of the terebra [42]; (2) in several species of the Braconidae, pre-apical ‘stop regions’ of the rhachis (e.g. swollen regions with scale-like sculptures located centrally within a corresponding widened part of the aulax at rest) increase friction if the 1st valvulae are retracted or extended thereby building up tension and compression and, thus, cause the terebra to curve because of the bending moment distribution [43] (cf. slide-lock working principle according to [104]); (3) in the braconid subfamily Doryctinae, a retraction of the 1st valvula causes the thinned outer walls of the aulaces to restrain the rhachis that features ancillary teeth, consequently resulting in a ventrad bending movement of the terebra [43]; (4) in the braconid genus *Zaglyptogastra*, the distal part of the terebra is formed into multi-arched and unevenly sclerotized regions, with the intermodal arched sections being more heavily sclerotized than the thinner nodes, and thus the protrusion of the 1st valvulae causes a flattening out of the nodal regions and the ventral flexing of the terebra [41]; (5) in several species of the Ichneumonidae, a largely longitudinally divided 2nd valvula, which is fused only at the apex, might allow the terebra to bend left or right when one part of the 2nd valvula is retracted [27]. In all these active bending mechanisms, the extent of bending movement can be controlled by adjustment of the amplitude of pro-/retraction of the individual valvulae [62]. Most of these parasitoid wasps are able to bend their terebra both dorso–ventrally and laterally, since multilateral steering can be achieved by the interplay of at least three elements [62], or by a rotational movement occurring simultaneously with the bending movement.

Passive and active bending mechanisms can technically act simultaneously or sequentially within the same structure.

During the oviposition process, female *L. distinguendus* were observed actively to bend their terebra in the air (i.e. in a cavity within a substrate) in which passive bending mechanisms can be excluded. The wasps were also observed to be able to pro- and retract the 1st valvula simultaneously with the bending movements (Fig. 2i–l; Additional file 1, min. 0:30–1:10, 1:36–1:44, 3:18–3:34) and independently of the bending state of the 2nd valvula and thus the whole terebra. The 1st valvulae can be protracted far forward and be retracted to a certain degree without significantly changing the bending of the whole terebra (arrowheads; Fig. 2i, k, m–o). This implies that the friction forces between the valvulae, i.e. in the olistheter system, are low. It further implies that no ‘stop regions’ or similar significant mechanical interactions occur between the 1st and 2nd valvulae in *L. distinguendus*. Despite rigorous searches (with scanning electron (SEM) and confocal laser scanning microscope (CLSM)), neither apical ‘stop structures’ in the olistheter nor evidence of a cluster-like occurrence of resilin in the terebra of *L. distinguendus* have been found (Additional file 2). Therefore, we conclude that the active bending mechanisms (1)–(4) mentioned above are not relevant for the terebra bending movements of *L. distinguendus*. The bending mechanisms for lateral and dorso–ventral bending and the rotation of the terebra of *L. distinguendus* are discussed in the following.

**Lateral bending of the terebra**: During the oviposition process of *L. distinguendus*, the terebra is anchored in the substrate. In this active probing position (Fig. 2a, c–l), a contraction of the anterior 2nd valvifer-2nd valvula muscles cannot elevate the terebra back into the resting position. In this case, however, a contraction of one of the m-a-2vf-2vv (*F*_m-a-2vf-2vv_; Fig. 7b) presumably pulls the corresponding bulb and thus the longitudinally split and asymmetrically overlapping 2nd valvula dorsad along its longitudinal axis (arrow 10; Fig. 7b, c) because of the orientation of the muscle and the resulting direction of the force vector. Since the two halves of the 2nd valvula are fused at the apex (Figs. 3a and 4d, i, k), this movement causes the distal part of the terebra (the part inside the cavity within the substrate) to bend to the left or right (Fig. 2i–l; Additional file 1, min. 1:02–1:10, 1:36–1:44): a contraction of the left m-a-2vf-2vv causes the 2nd valvula and thus the whole terebra to bend to the left (arrow 11; Fig. 7b, c; Table 1), whereas a contraction of the right m-a-2vf-2vv causes a bend to the right. The m-a-2vf-2vv in *L. distinguendus* is thus adapted to its lateral bending function of the terebra and no longer serves mainly as its elevator. Furthermore, in the active probing position, a simultaneous contraction of the paired posterior 2nd valvifer 2nd valvula muscles (*F*_m-p-2vf-2vv_; Fig. 7b) could also move the two overlapping halves of the 2nd valvula tangentially towards each other. However, the extent to which the resulting partial deformation of the 2nd valvula potentially allows local bending needs to be further investigated.

**Dorso–ventral bending of the terebra**: Female *L. distinguendus* can protract their 1st valvulae far beyond the apex of the 2nd valvula. However, these movements do not cause a dorsad bending movement of the terebra, indicating that no structures in the olistheter impede the movements of the 1st and 2nd valvulae relative to each other. However, a simultaneous retraction of both the 1st valvulae has been postulated to place the terebra under unilateral tension causing the apex to bend ventrad, and a retraction of a single 1st valvula causing the terebra to bend ventrad right or ventrad left [22, 25].

**Rotation of the terebra**: In the active probing position with the terebra being anchored in the substrate, a contraction of one of the posterior 2nd valvifer-2nd valvula muscles (*F*_m-p-2vf-2vv_; Fig. 7b) presumably causes the base of the 2nd valvula and thus the whole terebra to rotate at the basal articulation along its longitudinal axis to a certain degree. Even terebra rotations of up to 90° have been observed (Fig. 2n, o; Additional file 1, min. 1:45–1:54), although such extreme rotations are in part attributable to movements of the whole metasoma. Because of the orientation of the muscle in the active probing position and the resulting direction of the force vector, a contraction of the left m-p-2vf-2vv causes the 2nd valvula and thus the terebra to rotate anti-clockwise when viewed from the dorsal side (arrow 12; Fig. 7b, c; Table 1), whereas a contraction of the right m-p-2vf-2vv causes a rotation in a clockwise direction. Contraction of the m-a-2vf-2vv might further support these rotational movements. Alternating contractions of the left and right m-a-2vf-2vv and m-p-2vf-2vv cause a reciprocal rotation of the terebra, as can be observed during substrate penetration and drilling (cf. [25, 36, 60]). A rotation occurring simultaneously with lateral bending movements of the terebra allows the bending to become effective in various directions. The morphological structure of the basal articulations is well adapted for rotational movements (cf. [20]). Since the tendon of m-p-2vf-2vv runs over the curved dorsal side of the bulbous proximal end of the 2nd valvula, the effective inlever will presumably only change little over the range of motion. However, angular changes have a large impact on the torque that can be generated (cf. [32]) and on the resulting rotation. This mechanism of terebra rotation has also been postulated for other pteromalid [22], chalcidid [36] and aphelinid species [25].

Terebra bending movements in *L. distinguendus* do not result from mechanical interactions between the 1st and 2nd valvulae (as postulated for some species of Aulacidae and Gasteruptiidae [42] and Braconidae [41, 43]⁠). However, the slide-lock working principle (cf. [104]) is attained in a different way. The mechanism relevant for terebra bending in *L. distinguendus* shows similarities with that postulated for species of ophioniform Ichneumonidae, which feature a largely split 2nd valvulae that, like the one of *L. distinguendus*, is fused at the apex only [27, 39]. In these ichneumonid wasps, the pteromalid *L. distinguendus* and possibly also other chalcidoid taxa (see subchapter ‘Eco-evolutionary significance of terebra movements’ below), the terebra bending is presumably initiated by a bending of the 2nd valvula solely. The 1st valvulae, which are connected to it via the olistheter, can thus be pro- and retracted to a certain degree without significantly changing the bending state of the 2nd valvula and thus the whole terebra. This can be advantageous, e.g. for the penetration of the host larva’s skin for precise oviposition, whereby, in a bent state of the terebra, often alternating pro- and retraction movements of the 1st valvulae are required.

Whenever the 2nd valvula is bent in the lateral plane, one side (and thus also the lateral side of the corresponding 1st valvula) is under compression, with the opposite side being under tension. Both the bending and torsional stiffness of the terebra depend on its geometry, i.e. its cross-sectional shape (cf. [62]), and its material composition, i.e. chitin embedded in a protein matrix of variable mechanical properties (depending on its contents of resilin, arthropodin and sclerotin). The material stiffness of insect cuticle, expressed as Young’s modulus, has previously been estimated to range between 0.5–20 GPa [60, 81, 112].

### Eco-evolutionary significance of terebra movements

The structure of the terebra of Chalcidoidea, featuring a longitudinally split 2nd valvula with overlapping, asymmetric halves, is strongly consistent in structure within families and basically similar across families (with the exception of the primitive Mymaridae [18, 39], which recently have been identified as a sister group to all remaining Chalcidoidea [10, 49]), but is unique among other superfamilies of parasitoid Hymenoptera [39]. The similar structure of the terebra of chalcidoid taxa might indicate similar underlying working mechanisms, since form and function are strongly connected [113, 114]. Other chalcidoids are therefore also likely to be able to steer their terebra in a similar manner to that of *L. distinguendus*, as such terebra steering movements have also been observed in other species of Pteromalidae during the assessment of a potential host and egg placement [40, 46], in Eurytomidae during egg placement [21], in Eupelmidae during the assessment of a potential host [115] and the ovicide or larvacide of the competitors’ eggs and larvae, respectively [116], in species of the Aphelinidae during the ovicide of the competitors’ eggs [117–119] and in species of Torymidae [110] and Agaonidae for accurate egg deposition in the plant substrate [81, 106, 111] (although the latter two groups probably use passive bending mechanisms, unlike *L. distinguendus*).

Oviposition is crucial for the reproductive success of insects; thus, oviposition behaviour and ovipositor structure have a central adaptive role [83, 90, 93, 106, 120, 121] that should directly affect fitness. The improved manoeuvrability of the metasoma of the Apocrita, which is essential in the female wasp’s probing behaviour when searching or assessing a potential host, is attributed to the evolution of the wasp waist (a construction between the 1st and 2nd abdominal segment). The presence of a waist was a major innovation in the evolution of Hymenoptera and presumably contributed to the rapid diversification of Apocrita, since it allowed the successful attack of a variety of new hosts [3–5]. However, some chalcidoid wasps, e.g. species of Trichogrammatidae [122], have secondarily lost their wasp waist, presumably during miniaturization. Moreover, the vast majority of Chalcidoidea, although targeting the largest diversity of host taxa among parasitoid wasps [12], are idiobiont ectoparasitoids that develop on enclosed host stages with reduced mobility. Depositing eggs within a substrate provides them and the hatched larvae with the protection of a concealed environment (but without being exposed to the host’s immune system, as are endoparasitoids). Thus, in most chalcidoid wasps, as in *L. distinguendus*, a manoeuvrable metasoma does not improve the ability to reach hosts hiding in concealed cavities in hard substrates, since the position of the terebra is anchored at the puncture site. Moreover, drilling is extremely energy- and time-consuming (drilling a hole through a seed grain accounts for approximately 15% of the daily energy budget in a female eupelmid *Eupelmus vuilleti* (Crawford, 1913) [123]) and risky, as the wasps are exposed to predators. In *L. distinguendus* and presumably most chalcidoids that parasitize hosts hidden in hard substrates, the ability actively to bend and rotate the terebra in various directions is crucial during the search for a potential host within the substrate (or the cavity within), a targeted venom injection (e.g. directly into the ganglia [124, 125] or fat bodies of large hosts [126]), the assessment of the potential host, the ovicide and larvicide of the competitors’ eggs and larvae, respectively, the search for a suitable place for oviposition and controlled egg placement (Fig. 7d).

In Chalcidoidea, multiple morpho-physiological and behavioural traits have evolved in correlation with the use of hosts concealed deep within hard substrates, apparently related to several functional demands including host localization, substrate penetration, oviposition and emergence from the substrate (cf. [14]). Modifications or specializations of these traits, such as the ability actively to steer the terebra, may have interacted synergistically to open up new evolutionary pathways (cf. [5]). Adaptations in oviposition behaviour combined with morphological modifications of the terebra and adaptations in the function of certain muscles (i.e. the anterior and posterior 2nd valvifer-2nd valvula muscles) have potentially facilitated the evolution of terebra steering mechanisms, which in turn have facilitated the acquisition of new hosts to a parasitoid’s host range. These shifts in host exploitation allow niche partitioning among co-occurring species (cf. [127]) and presumably have led to rapid adaptive radiations in Chalcidoidea [10] (for speciation process in *L. distinguendus* see [128–130]). Thus, the ability actively to steer the terebra potentially has been a central factor in the evolution of the parasitoid life history strategy and the diversification of chalcidoid wasps, resulting in the evolutionary success of this group with its tremendous extant species richness [10, 49]. The Chalcidoidea are the most diverse group of parasitoid hymenopterans, with estimations of more than 500,000 chalcidoid species, the vast majority of them being parasitoids, out of a total of 680,000 parasitoid hymenopteran species [6, 7]. Even larger species numbers might exist because of the possibility of a vast underlying biodiversity of cryptic species (cf. [7, 8, 11]).

## Conclusions

Adaptations in oviposition behaviour combined with morphological modifications of the terebra and adaptations in the function of certain muscles allow *L. distinguendus* and presumably also other chalcidoid wasps to steer their terebra in various directions, a crucial skill for the successful oviposition of hosts that are concealed in substrates. Therefore, the evolution of the ability actively to steer the terebra can be considered as a putative key innovation that has largely contributed to the acquisition of new hosts to a parasitoid’s host range. Here, we have identified the structural adaptations, i.e. the longitudinally split and asymmetrically overlapping halves of the 2nd valvula that are fused at the apex and the functional adaptions of its associated muscles, and the mechanisms behind these innovations. Further comparative studies are needed to reveal the way in which morpho-physiological, behavioural, ecological and life history traits have interacted during the evolution and resulted in the enormous radiation of Chalcidoidea.

The terebra of hymenopterans in general and chalcidoids in particular can also act as a suitable biological concept generator, with further investigations into this matter possibly being helpful in the development and the design of slender miniaturized man-made probing tools (cf. [131–134]) for curved steering and drilling.

## Methods

The *L. distinguendus* used in this study originate from the laboratory colonies of FuturA GmbH (Borchen, Germany) and Biologische Beratung GmbH (Berlin, Germany), where they were bred on the larvae of *Sitophilus oryzae* (Linnaeus, 1763) (Coleoptera: Curculionidae) that develop endophytically in grain of the common wheat *Triticum aestivum* L.. This host indicates that the *L. distinguendus* used in this study probably belong to the karyotype with 5 chromosomes (‘*Sitophilus* Clade 1’ sensu [129] or ‘GW-lineage’ sensu [130], respectively) of the *L. distinguendus* species complex that comprises at least two morphologically indistinguishable cryptic species [135]. The clade/lineage presumably evolved by a host shift from drugstore beetles (*Stegobium paniceum* (Linnaeus, 1758)) to weevils of the genus *Sitophilus* [129]. This shift was probably related to the ability to learn from host-related cues [128].

For lateral overview images, female wasps were imaged with a digital microscope of the type Keyence Digital Microscope VHX-7000 (Keyence Corporation, Osaka, Japan) by using focus stacking.

Images were processed (white/black balancing, cropping) with GIMP version 2.10.30 (https://www.gimp.org; RRID:SCR_003182). The schematic drawings were created with Inkscape version 1.1 (https://www.inkscape.org; RRID:SCR_014479).

### High-resolution videography

The oviposition process of *L. distinguendus* was recorded in an artificial two-part Plexiglas chamber. Each lower chamber element featured a notch (ca. 4 · 1 · 1 mm) at the upper side of the front. Each upper element also featured a notch (ca. 4 · 2 · 4 mm) positioned at the front and lying exactly on that of the lower element. A piece of blotting paper was clamped in between the two elements to divide the space created by the two notches into two compartments. This paper was placed in the breeding substrate of *S. oryzae* for several days before the recording trials for it to take on the hosts’ scent (faecal cues) and thus to trigger the wasps to attempt to oviposit. The two chamber elements were then fixed with screws. A female *L. distinguendus* was placed into the upper compartment and a *S. oryzae* larva in the lower. The front and upper openings of the chamber were subsequently each closed with a clean glass coverslip. The process of oviposition was filmed in a horizonal position by using a Nikon DSC D90 camera (Nikon Corporation, Tokyo, Japan) mounted on a Leica MZ 12.5 stereomicroscope (Leica Microystems GmbH, Wetzlar, Germany) and with two LED cold-light sources KL 300 LED (Schott AG, Jena, Germany) for sufficient illumination. The focus was adjusted manually.

### Scanning electron microscopy (SEM)

For scanning electron microscopy (SEM), we dissected the ovipositor from the genital chamber of ethanol-fixed animals by using fine forceps. Specimens were dehydrated in a graded ethanol (C_2_H_6_O) series (30, 50, 70, 80, 90, 95 and twice 100% for 30 min each concentration) and air-dried for at least one week in a desiccator with silica gel (Carl Roth GmbH & Co. KG, Karlsruhe, Germany). We mounted the samples with double-sided adhesive carbon tape onto stubs and sputter coated them with 19 nm pure gold (Au) using an Emitech K550X (Quorum Technologies Ltd, West Sussex, UK). Investigation and imaging were performed with a scanning electron microscope of the type Zeiss EVO LS 10 (Carl Zeiss Microscopy GmbH, Jena, Germany) and the software SmartSEM version V05.04.05.00 (Carl Zeiss Microscopy GmbH, Jena, Germany).

### Confocal laser scanning microscopy (CLSM) and wide-field epifluorescence microscopy (WFM)

For confocal laser scanning microscopy (CLSM), specimens preserved in 70% ethanol were transferred to glycerol, dissected and further stored in a glycerol (ChemWorld, Kennesaw, GA, USA) droplet on concave microscope slides. Specimens were imaged between two #1.5 coverslips with a confocal laser scanning microscope of the type Nikon A1R-HD (Nikon Corporation, Tokyo, Japan). We used three excitation wavelengths, namely 409, 487 and 560 nm, and three emission ranges, namely 435–470, 500–540 and 570–645 nm. The resulting image sets were assigned pseudo-colours that reflected the fluorescence spectra (blue, green and red, respectively). Volume-rendered micrographs and media files were created using Fiji [136] (https://imagej.net/Fiji; RRID:SCR_002285).

For wide-field epifluorescence microscopy (WFM), we dissected the ovipositor from freshly killed individuals and washed them in distilled water. We mounted them carefully onto cleaned microscope slides (76 mm · 26 mm, VWR International, Radnor, PA, USA), embedded them in glycerol (Croma-Pharma GmbH, Loebendorf, Austria) without staining for observation with an epifluorescence microscope of the type Zeiss Axio Imager M2 (Carl Zeiss Microscopy GmbH, Jena, Germany) equipped with an ORCA-Flash4.0 V2 Digital CMOS camera C11440-22CU (Hamamatsu Photonics K.K., Hamamatsu, Japan) and the software ZEN 2 pro (blue edition) (Carl Zeiss Microscopy GmbH, Jena, Germany). We used Plan-Apochromat objectives and the following wavelength filters: DAPI (blue, excitation 353 nm, emission 465 nm), ATTO488 (green, excitation 500 nm, emission 525 nm) and Cy5 (red, excitation 650 nm, emission 673 nm).

We superimposed both the CLSM and WFM images in order to show the autofluorescence of the cuticular structures in order to analyse their material composition. Cuticular structures that predominantly show blue autofluorescence are composed of high proportions of the soft and highly elastic rubber-like amorphous protein resilin [98], which has an autofluorescence at a narrow band around 415 nm wavelength [95], whereas cuticular structures that autofluoresce in green are chitinuous and non- or weakly sclerotized and those that exhibit red autofluorescence are heavily sclerotized [98, 137].

### Sample preparation, light microscopy (LM), transmission electron microscopy (TEM) and image processing

Each female *L. distinguendus* was anaesthetized with carbon dioxide (CO_2_) before its metasoma was removed and submersed in fixative solution containing 2.5% glutaraldehyde (C_5_H_8_O_2_) and 5% sucrose (C_12_H_22_O_11_) buffered with 0.1 M cacodylate (C_2_H_7_AsO_2_) buffer (pH 7.4). During this process, the samples were stored in the fixative in small glass vials held in an ice bath at approximately 4 °C for 12 h, following which they were rinsed three times in pre-chilled 0.1 M cacodylate buffer (pH 7.4) for 10 min. After a 4 h period of post-fixation in 1% osmium tetroxide (OsO_4_) solution buffered with 0.1 M cacodylate buffer (pH 7.4) in an ice bath, the samples were again rinsed three times in the same buffer. The subsequent steps were performed at room temperature. The samples were dehydrated through a graded ethanol (C_2_H_6_O) series (30, 50, three times 70, 75, 80, 85, 90, 95 and 100% for three times, 10 min each concentration), containing *en-bloc* staining by using a saturated solution of 70% ethanolic uranyl acetate (C_4_H_6_O_6_U) for 12 h. The fully dehydrated samples were then passed through two changes of 100% propylene oxide (C_3_H_6_O) for 1 h per change and then through increasing concentrations of Spurr low-viscosity embedding resin (Polysciences Inc., Warrington, PA, USA) in propylene oxide with C_3_H_6_O:Spurr ratios of 3:1, 1:1, 1:3 and 1:7 for 1 h per change and 100% Spurr for 17 h on a rotatory shaker. As a last incubation step, the samples were placed in fresh pure resin for embedment in silicon moulds and polymerized at 70°C for 8 h.

Semithin (600 nm) and ultrathin (60 nm) sections were cut perpendicularly to the terebra of *L. distinguendus* by using an ultramicrotome of the type Leica Ultracut UTC (Leica Microsystems GmbH, Wetzlar, Germany) equipped with a DiATOME histo Jumbo diamond knife (45° knife angle; DiATOME Ltd, Nidau, Switzerland) with a large boat for continuous serial semithin sectioning or a DiATOME ultra diamond knife (35° knife angle; DiATOME Ltd, Nidau, Switzerland) for ultrathin sectioning. We conducted two complete section series through the whole metasoma; one continuous series of semithin sections and one with consecutive alternating series of 20 semithin and 10 ultrathin sections. Microscope slides (76 mm · 26 mm, VWR International, Radnor, PA, USA) for the mounting of semithin serial sections were preliminary stored in a bath containing 96% ethanol and 25% ammonia (NH_3_) at a C_2_H_6_O:NH_3_ ratio of 9:1 for at least one week and finally cleaned and stored in distilled water shortly before use. Semithin serial section bands were directly mounted onto these slides and stained with toluidine blue (C_15_H_16_ClN_3_S) for 60 s on a hot plate at 80°C. After being rinsed with distilled water and dried, the stained sections were embedded in Euparal (Waldeck GmbH & Co. KG, Münster, Germany). Ultrathin sections were placed on Formvar-coated copper slot grids and post-stained with 2% ethanolic uranyl acetate and lead citrate according to Venable and Coggeshall [138] for 20 and 10 min, respectively.

To image the semithin sections, we used a light microscope of the type Zeiss Axioplan (Carl Zeiss Microscopy GmbH, Jena, Germany) equipped with a Nikon D7100 single-lens reflex digital camera (Nikon Corporation, Tokyo, Japan) and the software Helicon Remote version 3.6.2.w (Helicon Soft Ltd, Kharkiv, Ukraine) (for focus stacking Helicon Focus version 6.3.7 Pro; RRID:SCR_014462). After initial image processing (white balancing, colour contrasting, black and white converting, cropping) in Adobe Lightroom version 6.0 (Adobe Systems, San José, CA, USA), the image stack was calibrated with Fiji [136] (https://imagej.net/Fiji; RRID:SCR_002285), a distribution of the software ImageJ2 version 2.3.0/1.53f [139, 140] (https://imagej.net; RRID:SCR_003070), and imported to the plugin TrakEM2 [141] (RRID:SCR_008954). A preliminary least square rigid alignment followed by an elastic alignment of the image stack was performed using the ‘Elastic Stack alignment’ plugin [142] in order to create an aligned image stack.

To investigate and image the ultrathin sections, we used a transmission electron microscope of the type Philips/FEI Tecnai 10 (FEI Company, Hillsboro, OR, USA) operated at 80 kV equipped with a side-mounted Gatan Rio9 CMOS camera (Gatan Inc., Pleasanton, CA, USA) and the software Tecnai G^2^ User Interface version 2.1.5 (FEI Company, Hillsboro, OR, USA) and DigitalMicrograph version 3.32.2403.0 (Gatan Inc., Pleasanton, CA, USA), respectively.

### Synchrotron X-ray phase-contrast microtomography (SR-µCT) and image processing

Two metasomas of ethanol-fixed female *L. distinguendus* were dehydrated stepwise in ethanol and critical-point-dried by using a Polaron 3100 (Quorum Technologies Ltd, West Sussex, UK) to avoid shrinking artefacts by water loss during the tomography procedure. The anterior ends of the metasomas were glued onto plastic pins and mounted onto the goniometer head of the sample stage for tomography. Synchrotron X-ray phase-contrast microtomography (SR-µCT) [143] was performed at the beamline ID19 at the European Synchrotron Radiation Facility (ESRF; Grenoble, France) at 19.5 keV (wavelength 8 · 10^−11^ m). 6000 projections were recorded over a 180° rotation with an effective detector pixel size of 0.3 µm, and a corresponding field of view of 0.63 · 0.63 mm. The detector consisted of a 4.5 μm thick LSO:Tb (Tb-doped Lu_2_SiO_5_) single-crystal scintillator lens (magnification 20×, numerical aperture (NA) 0.4) coupled to a sCMOS-based camera type pco.edge 5.5 (Excelitas PCO GmbH, Kelheim, Germany) [144, 145]. The detector-to-sample distance was set to 10 mm. Two separate overlapping image stacks were acquired since the structures of interest were larger than the field of view. The sample was therefore repositioned in between the imaging procedure, resulting in a certain overlap of the two consecutive images. The 3D voxels datasets were reconstructed from 2D radiographs by using the filtered back-projection algorithm [146, 147] developed for parallel-beam tomography.

The two resulting tomograms were registered and calibrated with Fiji [136] (https://imagej.net/Fiji; RRID:SCR_002285) and further imported to the plugin TrakEM2 [141] (RRID:SCR_008954) for stitching and cropping. Export of the aligned image stack was performed using a custom script, allowing the export of 16bit image stacks from TrackEM. Subsequently, the resulting image stack was imported to Amira version 6.0 (FEI Company, Hillsboro, OR, USA; RRID:SCR_014305) to pre-segment the various elements of the ovipositor and the whole metasoma in the software’s segmentation editor by manually labelling every 25th virtual slice and assigning them to different ‘materials’. These labels served as the input for automated segmentation by using the Biomedical Image Segmentation App ‘Biomedisa’ [148] (https://biomedisa.org). After some minor manual corrections to the segmentation results of the ‘Biomedisa’ output by using Amira, we converted them into polygon meshes. We thereby applied some minor smoothing (unconstrained smoothing, smoothing extent: 2) and polygon reduction to create the final 3D model (surface mesh).

### Supplementary Information

**Additional file 1**. Video sequences of female *Lariophagus distinguendus* parasitizing larvae of *Sitophilus granarius* in an artificial film chamber (cf. Fig. 2b–p). The terebra bending and rotating movements during host assessment and the alternate movements of the paired 1st valvulae can be observed.

**Additional file 2**. Superimposed CLSM images of the ovipositor of *Lariophagus distinguendus* (dorsal view, cf. Fig. 3g, h).

**Additional file 3**. Animation of the aligned semithin sections through the terebra of *Lariophagus distinguendus* (from distal to proximal; cf. Fig. 4a–d). The jittering movements of the two halves of the 2nd valvula in the middle of the image stack result from section and compression artefacts.

**Additional file 4**. Animation of the rotating segmented 3D model of the musculoskeletal ovipositor system of *Lariophagus distinguendus* (cf. Figs. 5 and 6).

## Data Availability

All data supporting the conclusions of this article are included within the article and its additional files. The full resolution videos (Additional file 1, Additional file 2, Additional file 3, and Additional file 4) and the analysed raw datasets are available from the corresponding author on reasonable request.

## Appendix 1

See Table 2.

**Table 2:** Morphological terms relevant to the hymenopteran ovipositor system

Anatomical term (abbreviation)	Definition/concept	Synonyms commonly found in literature	URI
1st valvifer (1vf)	The area of the 1st valvifer-1st valvula complex that is proximal to the aulax, bears the 9th tergal condyle of the 1st valvifer and the 2nd valviferal condyle of the 1st valvifer and is connected to the genital membrane by muscle	1. Valvifer [29]; Coin [HAO]; Crosse [HAO]; Écaille du stylet [HAO]; Fulcral plate [17–25, 35–37]; Furcal plate [HAO]; Gonangulum, gonangula [26, 27, 149–152]; Gonocoxite 8 [153]; Gonocoxite XIII [33, 34]; Kidney plate [154]; Plaque triangulaire [50]; Stiletträger [155]; Supporting plate [HAO]; Treibbein [HAO]; Triangular plate [28, 121, 156]; Vorderer Valvifer [29]; Winkel [HAO]; Winkelplatte [155]; Winkelstück [HAO]	http://purl.obolibrary.org/obo/HAO_0000338
1st valvifer-1st valvula complex	The anatomical cluster that is composed of the sclerites that articulates with the 9th abdominal tergite and the 2nd valvifer		http://purl.obolibrary.org/obo/HAO_0002158
1st valvifer-2nd valvifer muscle	The ovipositor muscle that arises from the interarticular ridge of the 1st valvifer and inserts on the 2nd valvifer		http://purl.obolibrary.org/obo/HAO_0002189
1st valvifer-genital membrane muscle (m-1vf-gm)	The ovipositor muscle that arises from the posterior part of the 1st valvifer and inserts anteriorly on the genital membrane anterior to the T9-genital membrane muscle	Anterior tergosternal strictor muscle [34]	http://purl.obolibrary.org/obo/HAO_0001746
1st valvula (1vv)	The area of the 1st valvifer-1st valvula complex that is delimited distally by the proximal margin of the aulax	1. Valvula [29]; 1st gonopophyse [149, 151, 152]; 1st gonapophysis [HAO]; Bohrborste [HAO]; Gonapophysis 8 [153]; Gonapophysis VIII [33, 34]; Gräte [HAO]; Lame de l’aiguillon [HAO]; Lancet [28, 154, 157–159]; Lower valve [16, 26, 27, 42, 43, 85, 88, 90, 93, 160–162]; Première valve [50]; Sägeblatt [HAO]; Sägeplatte [HAO]; Saw [HAO]; Schieber [HAO]; Soie piquante [HAO]; Spicula [HAO]; Stachelgräte [HAO]; Stechborste [29, 45, 155]; Stilet [HAO]; Stylet [17–25, 35–38, 73]; Valvae I [121]; Ventral stylet [163, 164]; Ventral valve [39, 44, 55, 71, 81, 86, 105, 150, 165]; Ventral valvula [163, 164]; Vordere Gonapophyse [HAO]	http://purl.obolibrary.org/obo/HAO_0000339
2nd valvifer (2vf)	The area of the 2nd valvifer-2nd valvula-3rd valvula complex that is proximal to the basal articulation and to the processus musculares and articulates with the female T9	2.Valvifer [29]; 2nd gonocoxa [149–152]; Écaille latérale [HAO]; Gonocoxite 9 [26, 27, 153]; Gonocoxite IX [33, 34]; Hinterer Valvifer [29]; Inner plate [35–37]; ~ Inner ovipositor plate [17–20, 23–25]; Lateral lobes [38]; Oblong plate [28, 121, 156]; Oblonge Platte [29, 155]; Plaque oblongue [HAO]; Portion invaginale [HAO]; Runner plate [154]; Scheide Grundteil [HAO]; Scheidenplatte [HAO]; ~ Semicuricular sheet [19–22]; Untere Hälfte der Seitenwand [HAO]; Vordere Platte [HAO]	http://purl.obolibrary.org/obo/HAO_0000927
2nd valvifer-2nd valvula-3rd valvula complex	The area that is connected to the 9th tergite and the 1st valvifer via conjunctiva, is articulated to the 1st tergite, and bears the aulax		http://purl.obolibrary.org/obo/HAO_0002175
2nd valvifer-3rd valvula complex	The area of the 2nd valvifer-2nd valvula-3rd valvula complex that is proximal to the basal articulation		http://purl.obolibrary.org/obo/HAO_0002181
2nd valvifer-genital membrane muscle	The ovipositor muscle that arises anteriorly from the dorsal flange of the 2nd valvifer and inserts anteriorly on the dorsal part of the genital membrane		http://purl.obolibrary.org/obo/HAO_0001672
2nd valvifer-venom gland reservoir muscle*	The muscle that arises from the 2nd valvifer and inserts on the venom gland reservoir		http://purl.obolibrary.org/obo/HAO_0002588
2nd valviferal condyle of the 1st valvifer	The condyle that is located on the 1st valvifer and articulates with the 1st valviferal fossa of the 2nd valvifer		http://purl.obolibrary.org/obo/HAO_0002167
2nd valvula (2vv)	The area of the 2nd valvifer-2nd valvula-3rd valvula complex that is distal to the basal articulation and to the processus musculares and is limited medially by the median body axis	2. Valvula [29]; 2nd gonapophyse [149]; 2nd gonapophysis [HAO]; Acus [HAO]; Back piece [HAO]; Branche et tige [HAO]; Corps tige et deux branches [HAO]; Deuxième valve [50]; Dorsal stylet [163, 164]; Dorsal valve [39, 43, 44, 71, 81, 86, 105, 150, 165]; Dorsal valvula [163, 164]; Fused 2nd gonopophyses [151, 152]; Gonapophysis 9 [153]; Gonapophysis IX [33, 34]; Gorgeret [HAO]; Gouttière lamellaire [HAO]; Internal sheath [HAO]; Lance [157]; Median (dorsal) valve [55]; Mittlere Gonapophysen [HAO]; Ovipositor sheath [35]; Ovipositor stylet [HAO]; Schienenrinne [29, 155]; Sheath [17, 18, 25, 38, 73]; Stachelrinne [29, 45]; Stachelschiene [HAO]; Sting shaft [158]; ~ Stylet (= slender distal part of the united 2nd valvulae) [28, 159]; Stylet sheath [19–24, 36, 37]; Upper valve [16, 26, 27, 42, 43, 85, 88, 90, 93, 160–162]; Valvae II [121]; (Fused) ventral valves [154]	http://purl.obolibrary.org/obo/HAO_0000928
3rd valvula (3vv)	The area of the 2nd valvifer-3rd valvula complex that is posterior to the distal vertical conjunctiva of the 2nd valvifer-3rd valvula complex	3. Valvula [29]; Articulating palp [22]; Beborstetes distales Ende der Scheidenplatte [HAO]; Dorsal valve [154]; Fourreau [HAO]; Gaine de l’aiguillon [HAO]; Gonoplac [149–152]; Gonostylus [153, 158]; Gonostylus IX [33, 34]; Hintere Gonapophyse [HAO]; Hüllschuppen [HAO]; Klappen [HAO]; Legescheiden [HAO]; Outer sheath [HAO]; Ovipositor sheath [16, 26, 27, 39, 160, 163–165]; Palp [19, 20, 36]; Palp-like appendage [35]; Palp-like termination of the inner plate [35]; Portion évaginale [HAO]; Sensory palp [25, 37]; Sheath [32, 43, 44, 105, 157]; Sheath lobe [28]; Sheath of saw [HAO]; Scheide [HAO]; Scheidenklappe [HAO]; Scheidenspitze [HAO]; Stachelscheide [29, 45, 155]; Stacheltaster [HAO]; Terminal palp [23]; Troisième valve [50]; Valvae III [121]; Valve [HAO]; Valve de fourreau [HAO]; Valve de la gaine du gorgeret [HAO]; Valve du fourreau [HAO]	http://purl.obolibrary.org/obo/HAO_0001012
7th abdominal sternum-vagina muscle*	The muscle that is attached to vagina and to the 7th abdominal sternum	Vaginal muscle [35]	http://purl.obolibrary.org/obo/HAO_0002595
9th tergal condyle of the 1st valvifer	The condyle that is located on the 1st valvifer and articulates with the 1st valviferal fossa of T9		http://purl.obolibrary.org/obo/HAO_0002160
Abdomen	The tagma that is located posterior to the thorax	Hinterleib [29, 45, 155]	http://purl.obolibrary.org/obo/HAO_0000015
Abdominal segment	The segment that is located posterior to the head and does not bear legs		http://purl.obolibrary.org/obo/HAO_0000016
Abdominal segment 7	The abdominal segment that is located between abdominal segment 6 and abdominal segment 8	5th gastral segment [HAO]; 6th metasomal segment [HAO]; 7th abdominal segment [HAO]; Abdominal segment VII [HAO]; Pygidium [HAO]; Segmentum abdominale septimum [HAO]; Segmentum septimum [HAO]	http://purl.obolibrary.org/obo/HAO_0000031
Abdominal segment 8	The abdominal segment that is located between abdominal segment 7 and abdominal segment 9	6th gastral segment [HAO]; 7th metasomal segment [HAO]; 8th abdominal segment [HAO]; Abdominal segment VIII [HAO]; Abdominalsegment 8 [29]; Metasomal segment 7 [HAO]; Segmentum abdominale octavum [HAO]; Segmentum octavum [HAO]	http://purl.obolibrary.org/obo/HAO_0000033
Abdominal segment 9	The abdominal segment that is located between abdominal segment 8 and abdominal segment 10	7th gastral segment [HAO]; 8th metasomal segment [HAO]; 9th abdominal segment [HAO]; Abdominal segment IX [HAO]; Abdominalsegment 9 [29]; Genital segment [HAO]; Hypopygium [HAO]; Metasomal segment 8 [HAO]; Segmentum abdominale nonum [HAO]; Segmentum nonum [HAO]; Tubular segment 9 [HAO]	http://purl.obolibrary.org/obo/HAO_0000034
Abdominal segment 10	The abdominal segment that is located posterior to abdominal segment 9	8th gastral segment [HAO]; 9th metasomal segment [HAO]; 10th abdominal segment [HAO]; Abdominal segment X [HAO]; Post-genital segment [166]; Segmentum abdominale decimum [HAO]; Segmentum decimum [HAO]	http://purl.obolibrary.org/obo/HAO_0000018
Abdominal sternum	The sternum that is located on the abdominal segment		http://purl.obolibrary.org/obo/HAO_0001425
Abdominal sternum 7	The abdominal sternum that is located on abdominal segment 7	6. Bauchschuppe [HAO]; 7. Sternit [HAO]; 7th abdominal sternite [HAO]; 7th abdominal sternum [HAO]; 7th sternite [35]; 7th sternum [166]; Abdominal sternum 7 [HAO]; Abdominal sternum VII [HAO]; Hypopygium [HAO]; Letzte Bauchschuppe [HAO]; Metasomal sternum VI [HAO]; Plaque ventral [HAO]; Sternum abdominale septimum [HAO]; Sternum septimum [HAO]; Subgenital plate [28, 166]; Subgenitalplatte [HAO]	http://purl.obolibrary.org/obo/HAO_0000044
Abdominal sternum 8	The abdominal sternum that is located on abdominal segment 8	8th abdominal sternite [HAO]; 8th abdominal sternum [HAO]; 8th sternite [35]; Abdominal sternite VIII [HAO]; Abdominal sternum VIII [HAO]; Genital plate [HAO]; Metasomal sternum VII [HAO]; Sternum abdominale octavum [HAO]; Sternum octavum [HAO]	http://purl.obolibrary.org/obo/HAO_0001531
Abdominal sternum 9	The abdominal sternum that is located on abdominal segment 9	9th abdominal sternite [HAO]; 9th abdominal sternum [HAO]; 9th sternal lobe [HAO]; 9th sternite [35]; Annular lamina [HAO]; Hypandrium [HAO]; Hypopygidium [HAO]; Hypopygium [HAO]; Hypotome [HAO]; Lamina subgenitalis [HAO]; Metasomal sternum 8 [HAO]; Metasomal sternum VIII [HAO]; Poculum [HAO]; Postgenital plate [HAO]; Sternum abdominale nonum [HAO]; Sternum nonum [HAO]; Subgenital plate [HAO]	http://purl.obolibrary.org/obo/HAO_0000047
Abdominal sternum 10	The abdominal sternum that is located on abdominal segment 10	10th sternite [35]	http://purl.obolibrary.org/obo/HAO_0001890
Abdominal tergum	The tergum that is located in the abdomen		http://purl.obolibrary.org/obo/HAO_0001426
Abdominal tergum 7	The tergum that is located on abdominal segment 7	5th gastral tergite [HAO]; 6th metasomal tergite [HAO]; 7th abdominal tergite [HAO]; 7th abdominal tergum [HAO]; 7th tergite [35]; Abdominal tergum VII [HAO]; Metasomal tergum VI [HAO]; Pypigidium [HAO]; Tergum abdominale septimum [HAO]; Tergum septimum [HAO]	http://purl.obolibrary.org/obo/HAO_0000060
Abdominal tergum 8	The tergum that is located on abdominal segment 8	6th gastral tergite [HAO]; 7. Rückensegment [HAO]; 7th metasomal hemitergite [158]; 7th metasomal tergite [HAO]; 7th metasomal tergum [HAO]; 8. Tergit [HAO]; 8º tergite [HAO]; 8th abdominal tergite [HAO]; 8th abdominal tergum [HAO]; 8th hemitergite [HAO]; 8th tergite [35]; 8th tergum [28]; Abdominal terga 8 [HAO]; Abdominal tergite VIII [HAO]; Epigynum [HAO]; Epipygium [HAO]; Lamella basalis superior [HAO]; Metasomal tergum VII [HAO]; Plaque oblongue [HAO]; Plaque stigmatifère [HAO]; Plaque stigmatique [HAO]; Spiracle (plate) [20, 22, 23, 25]; Spiracular plate [28]; Stigmenplatte [HAO]; T8 [HAO]; Tergite 8 [HAO]; Tergitplatte [HAO]; Tergum abdominale octavum [HAO]; Tergum octavum [HAO]; Trachealplatte [HAO]; Tracheenplatte [HAO]	http://purl.obolibrary.org/obo/HAO_0000061
Abdominal tergum 9	(cf. Female T9)	(cf. Female T9)	(cf. Female T9)
Abdominal tergum 10	The tergum that is located on abdominal segment 10	8th gastral tergite [HAO]; 8th gastral tergum [HAO]; 9th metasomal tergite [HAO]; 9th metasomal tergum [HAO]; 10th abdominal tergite [HAO]; 10th tergite [35]; Abdominal tergum X [HAO]; Epipygium [HAO]; Tergum abdominale decimum [HAO]; Tergum decimum [HAO]	http://purl.obolibrary.org/obo/HAO_0000052
Accessory gland	The gland that empties into one of the reproductive ducts		http://purl.obolibrary.org/obo/HAO_0000078
Acellular anatomical structure	Anatomical structure that consists of cell parts and cell substances and together does not constitute a cell or a tissue		http://purl.obolibrary.org/obo/HAO_0000040
Anatomical cluster	The anatomical group that has its parts adjacent to one another		http://purl.obolibrary.org/obo/HAO_0000041
Anatomical entity	Biological entity that is either an individual member of a biological species or constitutes the structural organization of an individual member of a biological species		http://purl.obolibrary.org/obo/HAO_0000000
Anatomical group	Anatomical structure consisting of at least two non-overlapping organs, multi-tissue aggregates or portion of tissues or cells of different types that does not constitute an organism, organ, multi-tissue aggregate, or portion of tissue		http://purl.obolibrary.org/obo/HAO_0000054
Anatomical region	A 3D region in space without well-defined compartmental boundries; for eample, the dorsal region of an ectoderm		http://purl.obolibrary.org/obo/HAO_0001980
Anatomical structure	Material anatomical entity which has an inherent 3D shape and is generated by coordinated expression of the organism’s own genome		http://purl.obolibrary.org/obo/HAO_0000003
Anatomical system	Anatomical group that has as its parts distinct anatomical structures interconnected by anatomical structures at a lower level of granularity		http://purl.obolibrary.org/obo/HAO_0000011
Annulus	The carina that is transverse and extends across the lateral wall of the terebra		http://purl.obolibrary.org/obo/HAO_0001173
Anterior	Anatomical region anteriorly located on the body or body part		http://purl.obolibrary.org/obo/BSPO_0000071
Anterior 2nd valvifer-2nd valvula muscle (m-a-2vf-2vv)	The ovipositor muscle that arises from the anterodorsal part of the 2nd valvifer and inserts subapically on the processus articularis	Anterior gonocoxapophyseal muscle [33, 34]; Gonapophysis 9 levator [153]; Ramus muscle of the 2nd valvula [28]; Ramus muscle of the 2nd valvulae [166]; Shaft elevator muscle [25]; Sting levator [156]	http://purl.obolibrary.org/obo/HAO_0001166
Anterior angle of the 1st valvifer	The corner on the 1st valvifer that marks the posterior end of the 1st valvula		http://purl.obolibrary.org/obo/HAO_0002168
Anterior area of the 2nd valvifer	The area of the 2nd valvifer which is anterior to the anatomical line that is the shortest distance from the 1st valviferal fossa of the 2nd valvifer and the ventral margin of the 2nd valvifer		http://purl.obolibrary.org/obo/HAO_0002169
Anterior flange of abdominal tergum 9	The flange that extends along the anterolateral margin of female T9	Apodem [29]; Chitinous rib [37]; Chitinous rib of the outer plate [35]	http://purl.obolibrary.org/obo/HAO_0001171
Anterior flange of the 1st valvifer	The flange that extends anteriorly on the 1st valvifer and overlaps with the posterior margin of the anterior area of the 2nd valvifer		http://purl.obolibrary.org/obo/HAO_0002166
Anterior notch of the dorsal valve	The notch that is on the anterior region of the dorsal valve (= composite structure of the fused 2nd valvulae) and accommodates the ventral ramus of the 2nd valvula and the 1st valvula		http://purl.obolibrary.org/obo/HAO_0002178
Anterior ridge of T9	The ridge that extends along the anterior margin of female T9 and receives the site of origin of the ventral and the dorsal T9-2nd valvifer muscles		http://purl.obolibrary.org/obo/HAO_0002182
Anterior section of dorsal flange of the 2nd valvifer	The area of the anterior area of the 2nd valvifer that is anterior to the site of origin of the basal line, continuous with the dorsal flange of the 2nd valvifer and marks the site of origin of the dorsal T9-2nd valvifer muscle part b	~ Falcate plate [35]; ~ Pivoting sclerite [36]; ~ Semicircular sheet [19–25]	http://purl.obolibrary.org/obo/HAO_0002173
Apical	Anatomical region located on the apical end on the body or body part	Distal [BSPO]	http://purl.obolibrary.org/obo/BSPO_0000073
Apodeme	The process that is internal		http://purl.obolibrary.org/obo/HAO_0000142
Aporous sensillum	The sensillum whose cuticular component is without any opening		http://purl.obolibrary.org/obo/HAO_0001964
Appendage	The anatomical structure that is encircled by the evagination of the integument and whose integumental components are attached to the body via muscles		http://purl.obolibrary.org/obo/HAO_0000144
Area	The anatomical structure of the cuticle that is delimited by material or immaterial anatomical entities	Potion of cuticle [HAO]	http://purl.obolibrary.org/obo/HAO_0000146
Articular surface	The area that is located on the sclerite and that makes movable direct contact with another sclerite	Articulation [HAO]	http://purl.obolibrary.org/obo/HAO_0001485
Articulation	The anatomical cluster that is composed of two adjacent articular surfaces		http://purl.obolibrary.org/obo/HAO_0000151
Aulax (au)	The impression that is on the 1st valvifer-1st valvula complex accommodates the rhachis	Falze der Stechborste [155]; Groove [17, 19, 20, 22, 25, 36, 37, 166]	http://purl.obolibrary.org/obo/HAO_0000152
Basal	Anatomical region located basally on the body or body part	Proximal [BSPO]	http://purl.obolibrary.org/obo/BSPO_0000074
Basal articulation (ba)	The articulation that is part of the 2nd valvifer-2nd valvula-3rd valvula complex and adjacent to the rhachis	Basalgelenk [29]; Bulbous articulation [17, 19, 20, 22, 24, 25, 153]	http://purl.obolibrary.org/obo/HAO_0001177
Basal line of the 2nd valvifer	The line on the 2nd valvifer that extends between the pars articularis and the dorsal flange of 2nd valvifer		http://purl.obolibrary.org/obo/HAO_0002171
Body	The anatomical cluster that is composed of the whole organism but which excludes the antennae, legs and wings	Trunk [HAO]	http://purl.obolibrary.org/obo/HAO_0000182
Bulb (blb)	The anterior area of the dorsal valve (= composite structure of the fused 2nd valvulae) that is bulbous	Backen [155]; Bulbous basal part of the united 2nd valvulae [28]; Bulbous sockets [20, 22, 24]; Pivoting process [36]; Rotatory process [36, 37]; Sockets [25]	http://purl.obolibrary.org/obo/HAO_0002177
Calyx*	The broadened portion of the lateral oviduct is enlarged and contains virus particles (characterized by bluish autofluorescence)	Calyx gland [26]; Eierkelch [HAO]	http://purl.obolibrary.org/obo/HAO_0000186
Captivated compound organ	Compound organ that contains one or more macroscopic spaces		http://purl.obolibrary.org/obo/HAO_0000072
Carina	The process that is elongate and external	Crest [HAO]; Lamella [HAO]; Lamina [HAO]; Ledge [HAO]; Ridge [HAO]	http://purl.obolibrary.org/obo/HAO_0000188
Cell	Anatomical structure that has its parts a maximally connected cell compartment surrounded by a plasma membrane		http://purl.obolibrary.org/obo/HAO_0000013
Cercus	The sense organ that is located apicolaterally on one of the apicalmost terga and attaches to a large nerve cord	Acrocercus [HAO]; Appendix genitalis [HAO]; Circus [36]; Penicillus [HAO]; Pygopod [HAO]; Pygostyle [20, 22–24]; Pygostylus [HAO]; Sensory appendage [37]; Sensory plate [35, 37]; Socius [29]	http://purl.obolibrary.org/obo/HAO_0000191
Colleterial gland	The glandula mucosa that secretes an adhesive substance used to fasten the eggs to a support	Accessory gland [72, 73]; Collaterial gland [27]; Glande accessoire [50]; Glandula mucosa [HAO]; Lubricating gland [72]; Uterine gland [27]; Uterus gland [26]; Vaginal gland [27]	http://purl.obolibrary.org/obo/HAO_0000214
Common oviduct (co)*	The duct that is unpaired and connects the lateral oviducts with the vagina through the gonopore	Median oviduct [HAO]; ~ Vagina [20, 22, 23, 25, 73, 75]	http://purl.obolibrary.org/obo/HAO_0002601
Compound organ	Anatomical structure that has as its parts two or more multi-tissue structures of at least two different types and which through specific morphogenetic processes forms a single distinct structural unit demarcated by bona fide boundaries from other distinct anatomical structures of different types		http://purl.obolibrary.org/obo/HAO_0000024
Condyle	The articular surface that is convex and is inserted into the fossa of an adjacent sclerite		http://purl.obolibrary.org/obo/HAO_0000220
Conjunctiva	The area of the cuticle that is weakly sclerotized, with thin exocuticle	Arthropodial membrane [HAO]; Corium [HAO]	http://purl.obolibrary.org/obo/HAO_0000221
Cordate apodeme	The apodeme on the anterior margin of female T9. The ventral T9-2nd valvifer muscle attaches partly on the apodeme	Apophyse [29]	http://purl.obolibrary.org/obo/HAO_0001585
Corner	The projection that is located at the intersection of two or more edges		http://purl.obolibrary.org/obo/HAO_0000223
Ctenidium (ct)	The process that is located on the annulus	~ Oblique ridge [86]; (Ovipositor) scales [71]	http://purl.obolibrary.org/obo/HAO_0001190
Cuticle	The acellular anatomical structure that is the external layer of the integument (covers a body surface as well as lines ectodermal invaginations such as stomodeum, proctodeum and tracheae) and produced by epidermis cells	Cuticula [HAO]; Exoskeleton [HAO]	http://purl.obolibrary.org/obo/HAO_0000240
Cuticular invagination	The area where the cuticle is invaginated	Invagination [HAO]	http://purl.obolibrary.org/obo/HAO_0002021
Depression	The area that is external, concave, point-like and does not correspond to an apodeme	Fovea [HAO]; Pit [HAO]; Trough [HAO]	http://purl.obolibrary.org/obo/HAO_0000241
Distal notch of the dorsal valve	The notch that is distal on the dorsal valve (= composite structure of the fused 2nd valvulae)		http://purl.obolibrary.org/obo/HAO_0002179
Distal vertical conjunctiva of the 2nd valvifer-3rd valvula complex	The conjunctiva that traverses the 2nd valvifer-3rd valvula complex and is located distal to the median bridge of the 2nd valvifers		http://purl.obolibrary.org/obo/HAO_0002180
Dorsal	Anatomical region dorsally located on the body or body part		http://purl.obolibrary.org/obo/BSPO_0000079
Dorsal 2nd valvifer-venom gland reservoir muscle (m-d-2vf-vr)*	The 2nd valvifer-venom gland reservoir muscle that originates at the medial surface of the anterior part of the 2nd valvifer and inserts dorsally at the anterior part of the venom gland reservoir		http://purl.obolibrary.org/obo/HAO_0002597
Dorsal flange of the 2nd valvifer	The flange that extends on the dorsal margin of the 2nd valvifer. Part of the ventral T9-2nd valvifer muscle attaches to the flange	Chitinous rib of the inner plate [35]; Dorsale Verdickungsleiste [29]; Inner longitudinal rib (of the inner plate) [37]	http://purl.obolibrary.org/obo/HAO_0001577
Dorsal projection of the 2nd valvifer (dp2)	The projection that is located on the 2nd valvifer and corresponds to the proximal end of the rhachis	~ Ramus edge [19–22, 25]	http://purl.obolibrary.org/obo/HAO_0002172
Dorsal ramus of the 1st valvula (dr1)	The region that extends along the dorsal margin of the 1st valvula and bears the aulax	1st ramus [167]; Arc chitineux [50]; Diverging arm of the stylet [35]; Ramus der 1. Valvula [29]; Ramus of the 1st gonapophysis [150]; Ramus of the 1st valvula [28, 168]; Stechborstenbogen [29]	http://purl.obolibrary.org/obo/HAO_0001579
Dorsal ramus of the 2nd valvula	The area that extends along the dorsal margin of the 2nd valvula, bears the processus articularis anteriorly and the processus musculares on the antero-dorsal region and articulates with the 2nd valvifer via the basal articulation	2nd ramus [167]; Diverging arm of the ovipositor sheath [35]; Ramus der 2. Valvula [29]; Ramus of the 2nd gonapophysis [150]; Schienenbogen [29, 155]	http://purl.obolibrary.org/obo/HAO_0002190
Dorsal sclerite of the 1st valvifer	The sclerite of the 1st valvifer that is located dorsally of the transvalviferal conjunctiva		http://purl.obolibrary.org/obo/HAO_0002163
Dorsal T8-T9 muscle	The abdominal muscle that arises from the anteromedian margin of female T8 and inserts on the anteromedian margin of female T9		http://purl.obolibrary.org/obo/HAO_0001571
Dorsal T9-2nd valvifer muscle (m-d-T9-2vf)	The ovipositor muscle that arises along the posterodorsal part of the anterior margin of female T9 and inserts on the anterior section of the dorsal flanges of the 2nd valvifer	Anterior tergal muscle [166]; Anterior tergogonocoxal muscle [33, 34]; Dorsal/ventral (= part a/part b) anterior tergal muscle of the 2nd valvifer [28]; Extensor muscles of lancet [154]; Upper/lower (= part a/part b) protractor of gonapophysis 8 [153]; Upper/lower (= part a/part b) stylet protractor muscle [25]	http://purl.obolibrary.org/obo/HAO_0001569
Dorsal valve	The area that is articulated with the right and left 2nd valvifers at the basal articulation and bears the rhachises. (Term sometimes used for the composite structure of the fused 2nd valulae.)	(cf. 2nd valvula)	http://purl.obolibrary.org/obo/HAO_0001658
Duct	The captivated compound organ that is canal-like, layered with epithelial cells and leads to an exocrine gland or organ		http://purl.obolibrary.org/obo/HAO_0000282
Dufour’s gland (Dg)*	The accessory gland that is not paired and empties into the female reproductive duct and is composed of class III gland cells	Alkaline gland [19–25, 73]; Glande alkaline [50]; Lubricating gland [89]; Tubular gland [73]	http://purl.obolibrary.org/obo/HAO_0002591
Dufour’s gland duct (Dgd)*	The duct that is the proximal-most section of the Dufour’s gland between the gland orifice and the Dufour’s gland closing muscles	Duct of the acid gland [73]; Stalk (of the Dufour’s gland) [77, 79]	http://purl.obolibrary.org/obo/HAO_0002600
Edge	The margin that extends along the border of two areas that are oriented differently		http://purl.obolibrary.org/obo/HAO_0000285
Egg canal (ec)	The anatomical space that is between the left and right olistheters	Central canal [36]; Egg passage [35]; Eikanal [29]; Innenkanal [29, 155]	http://purl.obolibrary.org/obo/HAO_0002191
External female genitalia	The anatomical cluster that is composed of the vagina, spermatheca and the ovipositor apparatus		http://purl.obolibrary.org/obo/HAO_0001768
Female genitalia	The genitalia that is part of the female organism		http://purl.obolibrary.org/obo/HAO_0000324
Female T8	The tergite that is connected to female T9 by muscles	(cf. Abdominal tergum 8)	http://purl.obolibrary.org/obo/HAO_0002188
Female T9 (T9)	The tergite that is articulated with the 1st valvifer and is connected to the 2nd valvifer via muscles	8th metasomal hemitergite [158]; 9. Tergit [29]; 9° tergite [HAO]; 9th abdominal tergite [26, 32]; 9th hemitergite [HAO]; 9th tergite [35, 75, 157]; 9th tergum [28]; Abdominal tergum 9 [HAO]; Afterplatte [HAO]; Anal plate [HAO]; Anterior lobes [38]; Anterior plate [HAO]; Écaille anale [HAO]; Écaille chitineuse [HAO]; Epandrium [HAO]; Epipygium [HAO]; Épipygium [HAO]; Flattened plate [38]; Hintere Platte [155]; Hypopygial valve [HAO]; Lamine esterne [HAO]; Lateral plates [89]; Obere Hälfte der Seitenwand [HAO]; Outer ovipositor plate[17–25]; Outer plate (of ovipositor) [35–37]; Plaque carrée [50]; Plaque quadratique [HAO]; Quadrate plate [28, 121, 150, 156]; Quadratische Platte [29, 155]; Quadratplatte [HAO]; Sled plate [154]; T9 [27, 30, 32, 102, 168–170]; Terga 9 [163, 164]; Tergite 9 [83, 153]; Tergite IX [33, 34]; Tergum 9 [30, 168–171]; Tergum IX [167]; Tergum abdominale nonum [HAO]; Tergum nonum [HAO]	http://purl.obolibrary.org/obo/HAO_0000075
Flange	The projection that is lamella-like and is located on a rim, carina, apodeme or edge	Crest [HAO]	http://purl.obolibrary.org/obo/HAO_0000344
Foramen	The anatomical space that is surrounded by sclerites and allows for the passage of haemolymph, nerves and tracheae		http://purl.obolibrary.org/obo/HAO_0000345
Fossa	The articular surface that is concave and accommodates the condyle of another sclerite	Acetabulum [HAO]	http://purl.obolibrary.org/obo/HAO_0000353
Furcula (fu)	The sclerite that is proximal to the 2nd valvifer and receives the site of origin of the posterior 2nd valvifer-2nd valvula muscle	Chitinios fork of the vagina [36]; Gabelbein [29]; V-shaped sclerite [35]	http://purl.obolibrary.org/obo/HAO_0002498
Gaster	The anatomical cluster that is composed of all abdominal segments posterior to abdominal segment 2		http://purl.obolibrary.org/obo/HAO_0000369
Genital membrane	The conjunctiva that connects the ventral margins of the 2nd valvifers arching above the 2nd valvula		http://purl.obolibrary.org/obo/HAO_0001757
Germarium*	Anterior region of the ovariole, that is connected to posterior egg chambers		http://purl.obolibrary.org/obo/FBbt_00004866
Gland	The anatomical cluster that is composed of epithelial cell(s) that secrete or excrete materials not related to their ordinary metabolic needs		http://purl.obolibrary.org/obo/HAO_0000375
Gonopore*	The anatomical space that is the transition from the common oviduct to the vagina		http://purl.obolibrary.org/obo/HAO_0002602
Groove	The line that is located on the sclerite and is impressed		http://purl.obolibrary.org/obo/HAO_0001525
Hypopygium	The abdominal sternum that is the posteriormost visible sclerite located ventrally in the abdomen	Hypandrium [HAO]; Subgenital plate [HAO]	http://purl.obolibrary.org/obo/HAO_0000410
Immaterial anatomical entity	Anatomical entity that has no mass		http://purl.obolibrary.org/obo/HAO_0000007
Impression	The groove that does not correspond to a ridge	Groove [HAO]	http://purl.obolibrary.org/obo/HAO_0000417
Integument	The anatomical system that forms the covering layer of the animal, ectodermal in origin and composed of epidermal cells producing the cuticle	Body wall [HAO]	http://purl.obolibrary.org/obo/HAO_0000421
Interarticular ridge of the 1st valvifer (iar)	The ridge that extends along the posterior margin of the 1st valvifer between the intervalvifer and tergovalvifer articulations		http://purl.obolibrary.org/obo/HAO_0001562
Interlock of the 1st valvulae (il1)*	The anatomical cluster that consists of the apical regions of the left and right 1st valvulae, which are interlocked with each other in a manner similar to how the 1st and 2nd valvulae are connected by olistheter		http://purl.obolibrary.org/obo/HAO_0002603
Intervalvifer articulation (iva)	The articulation between the 1st valvifer and 2nd valvifer	Articulating process of the fulcral plate with the inner plate [35]; Intervalviferengelenk [29]	http://purl.obolibrary.org/obo/HAO_0001558
Intravalvifer articulation	The articulation between the dorsal sclerite of the 1st valvifer and the ventral sclerite of the 1st valvifer		http://purl.obolibrary.org/obo/HAO_0002165
Laminated bridge (lb)	The area that is located proximally on the notal membrane near the processus articularis, is sclerotized and is continuous with the 2nd valvula	Chitinstäbchen [29]; Chitinous cross striae uniting the rods of the sheath [35]; Transverse chitinous ribs [36, 37]	http://purl.obolibrary.org/obo/HAO_0001548
Lateral	Anatomical region laterally located on the body or body part		http://purl.obolibrary.org/obo/BSPO_0000082
Lateral T8-T9 muscle	The 9th abdominal tergal muscle that arises from the anterolateral margin of female T8 and inserts on the anterolateral margin of female T9		http://purl.obolibrary.org/obo/HAO_0001776
Lateral T9-2nd valvifer muscle	The muscle arises from the posteroventral parts of the female T9 and inserts on the median bridge		http://purl.obolibrary.org/obo/HAO_0002187
Line	The anatomical cluster that is linear		http://purl.obolibrary.org/obo/HAO_0001586
Lumen (Anatomical space)	Non-material entity of three dimensions, that is generated by morphogenetic or other physiologic processes; is surrounded by one or more anatomical structures; contains one or more organism substances or anatomical structures		http://purl.obolibrary.org/obo/HAO_0000005
Margin	The line that delimits the periphery of an area		http://purl.obolibrary.org/obo/HAO_0000510
	The anatomical region that extends along the margin		http://purl.obolibrary.org/obo/HAO_0001981
Medial	Anatomical region medially located on the body or body part		http://purl.obolibrary.org/obo/BSPO_0000083
Medial 2nd valvifer-2nd valvula muscle*	The muscle that arises anteromedially from the 2nd valvifer and inserts inside the lumen of 2nd valvula		http://purl.obolibrary.org/obo/HAO_0002596
Medial conjunctiva of the 1st valvulae	The conjunctiva that extends medially along the 1st valvula		http://purl.obolibrary.org/obo/HAO_0002192
Medial ridge of the 2nd valvifer (mr2)*	The ridge that is on the anterior section of the dorsal flange and serves as the site of insertion of T9-2nd valvifer muscle part b		http://purl.obolibrary.org/obo/HAO_0002594
Median bridge of the 2nd valvifers (mb2)	The area that connects posterodorsally the 2nd valvifers and is the site of attachment for the posterior T9-2nd valvifer muscle	Bridge (of the inner plates) [37]; Bridge of 2nd valvifer [HAO]; Transverse ligament [19]	http://purl.obolibrary.org/obo/HAO_0001780
Median conjunctiva of abdominal tergum 9	The conjunctiva of abdominal tergum 9 that has median and longitudinal	Brücke [29]	http://purl.obolibrary.org/obo/HAO_0002267
Metasoma	The tagma that is connected anteriorly to the metapectal-propodeal complex at the propodeal foramen and consists of abdominal segments		http://purl.obolibrary.org/obo/HAO_0000626
Metasomal segment	The abdominal segment that is located in the metasoma		http://purl.obolibrary.org/obo/HAO_0001969
Metasomal sternum	The abdominal sternum that is located in the metasoma		http://purl.obolibrary.org/obo/HAO_0001350
Metasomal tergum	The abdominal tergum that is located in the metasoma		http://purl.obolibrary.org/obo/HAO_0001349
Muscle	The portion of tissue that is composed of contractile fibres		http://purl.obolibrary.org/obo/HAO_0000641
Nervous system	The organ system that is composed or neurons and glial cells		http://purl.obolibrary.org/obo/HAO_0001732
Notal membrane (nm)	The conjunctiva that connects the medial margins of the 2nd valvulae	Elaterium [39, 86]; ~ Ligamentum [157]	http://purl.obolibrary.org/obo/HAO_0001733
Notch	The part of the margin of a sclerite that is concave		http://purl.obolibrary.org/obo/HAO_0000648
Olistheter (oth)	The anatomical cluster that is composed of the rhachis of the 2nd valvula and the aulax of the 1st valvula	Tongue and groove arrangement [150]; Tongue and groove mechanism [20]	http://purl.obolibrary.org/obo/HAO_0001103
Organ system	A division of the whole organism into specialized systems		http://purl.obolibrary.org/obo/HAO_0001599
Orifice of the venom gland reservoir (ovr)*	The anatomical space at the proximal-most end of the venom gland reservoir	Opening of the reservoir [73]	http://purl.obolibrary.org/obo/HAO_0002599
Ovariole, ovarioles*	An egg assembly line. Consists of a germarium at the anterior tip connected to a chain of egg chambers, each one more mature than the preceding, more anterior egg chamber in the chain. Each ovariole is encased in an ovarian sheath		http://purl.obolibrary.org/obo/FBbt_00004893
Ovary, ovaries*	The female gonad	Ovaire [50]	http://purl.obolibrary.org/obo/FBbt_00004865
Oviduct*	Duct of the female reproductive tract that connects the ovaries to the uterus [172, 173]. The oviduct consists of two lateral oviducts (each connected to an ovary) and one common oviduct, to which the lateral oviducts connect, and which itself connects to the uterus [172, 173]		http://purl.obolibrary.org/obo/FBbt_00004911
Ovipositor	The anatomical cluster that is composed of the 1st valvulae, 2nd valvulae, 3rd valvulae, 1st valvifers, 2nd valvifers and female T9	Legeapparat [29, 155]; Ovipositor mechanism [19, 20, 25]; Stachelapparat [29]; Sting [HAO]; Sting apparatus [158, 167, 168, 170]; Stinger [HAO]	http://purl.obolibrary.org/obo/HAO_0000679
Ovipositor apparatus	The anatomical cluster that is composed of the ovipositor, abdominal terga 8–10, abdominal sternum 7 and muscles connecting them		http://purl.obolibrary.org/obo/HAO_0001600
Ovipositor muscle	The abdominal muscle that inserts on the ovipositor		http://purl.obolibrary.org/obo/HAO_0001290
Pars articularis	The articular surface that is situated anteriorly on the ventral margin of the 2nd valvifer and forms the lateral part of the basal articulation	Pars articulares [HAO]	http://purl.obolibrary.org/obo/HAO_0001606
Patch	The area that is round and differs from surrounding regions in sculpture, setae, and/or pigmentation		http://purl.obolibrary.org/obo/HAO_0000704
Portion of tissue	Anatomical structure that consists of similar cells and intercellular matrix, aggregated according to genetically determined spatial relationships		http://purl.obolibrary.org/obo/HAO_0000043
Post-ramus flap (prf)*	The distal area of the dorsal projection of the 2nd valvifer that is widened and is flexibly connected to the proximal region of the dorsal projection of the 2nd valvifer	Post-ramus flap of the 2nd valvifer [HAO]; ~ Post-ramus extension [19, 25]	http://purl.obolibrary.org/obo/HAO_0002593
Posterior	Anatomical region posteriorly located on the body or body part		http://purl.obolibrary.org/obo/BSPO_0000072
Posterior 2nd valvifer-2nd valvula muscle (m-p-2vf-2vv)	The ovipositor muscle that arises posteroventrally from the 2nd valvifer and inserts on the processus musculares of the 2nd valvula	Gonapophysis 9 depressor [153]; Muscle of the furcula [28, 166]; Posterior gonocoxapophyseal muscle [33, 34]; Retractor of ventral valves [154]; Sting depressor [156]	http://purl.obolibrary.org/obo/HAO_0001815
Posterior area of the 2nd valvifer	The area of the 2nd valvifer that is posterior to the anatomical line that is the shortest distance from the valviferal fossa of the 2nd valvifer to the ventral margin of the 2nd valvifer		http://purl.obolibrary.org/obo/HAO_0002170
Posterior margin of 1st valvifer	The margin of the 1st valvifer that is posterior and extends between the intervalvifer articulation and the anterior angle of the 1st valvifer		http://purl.obolibrary.org/obo/HAO_0002159
Posterior section of dorsal flange of the 2nd valvifer	The area of the dorsal flange of the 2nd valvifer that is posterior to the site of origin of the basal line	~ Inner ovipositor plate [19–25]	http://purl.obolibrary.org/obo/HAO_0002174
Posterior T9-2nd valvifer muscle (m-p-T9-2vf)	The ovipositor muscle that arises medially from the posterodorsal part of female T9 and inserts on the median bridge of the 2nd valvifers	Dorsal/lateral tergogonostylar muscle [33, 34]; Posterior dorso–ventral muscle [154]	http://purl.obolibrary.org/obo/HAO_0001813
Posteroventral corner of 1st valvifer	The corner of the 1st valvifer that is adjacent to the intervalvifer articulation		http://purl.obolibrary.org/obo/HAO_0002239
Prearticular incisure	The notch that is located on the margin of the 2nd valvifer immediately anterior to the basal articulation of the 2nd valvula		http://purl.obolibrary.org/obo/HAO_0000799
Process	The area on the sclerite that is raised		http://purl.obolibrary.org/obo/HAO_0000822
Processus articularis	The process that extends laterally from the proximal part of the 2nd valvula and forms the median part of the basal articulation, and corresponds to the site of attachment for the anterior 2nd valvifer-2nd valvula muscle		http://purl.obolibrary.org/obo/HAO_0001704
Processus musculares	The apodeme that extends dorsally from the proximal part of the 2nd valvula to the genital membrane and receives the site of attachment of the posterior 2nd valvifer-2nd valvula muscle	Processus muscularis [HAO]	http://purl.obolibrary.org/obo/HAO_0001703
Proctiger	The area that is located around the anal opening posterior to the epipygium and hypopygium		http://purl.obolibrary.org/obo/HAO_0001827
Projection	The process that is located on an edge		http://purl.obolibrary.org/obo/HAO_0000829
Proximal	Anatomical region proximally located on the body or body part		http://purl.obolibrary.org/obo/BSPO_0000077
Region	The anatomical structure that is delimited by at least one immaterial anatomical entity. (Class obsolete.)		http://purl.obolibrary.org/obo/HAO_0000893
Reproductive system	The anatomical system that is involved in reproduction		http://purl.obolibrary.org/obo/HAO_0000895
Rhachis (rh)	The ridge that extends along the ventral surface of the 2nd valvula that is partially enclosed by the aulax	Bead for attaching stylet to rod of sheath [35]; Leiste der Schienenrinne [155]; Rail [166]; Rail guides [81]; T-ridge of ventral valve [154]; Tongue [19, 20, 22, 25]	http://purl.obolibrary.org/obo/HAO_0000898
Ridge	The apodeme that is elongate	Lamella [HAO]; Lamina [HAO]	http://purl.obolibrary.org/obo/HAO_0000899
Rim	The carina that extends along the margin or edge of a sclerite		http://purl.obolibrary.org/obo/HAO_0000900
S7-1st valvula muscle	The muscle that originates from the abdominal tergum 7 and inserts on the 1st valvula		http://purl.obolibrary.org/obo/HAO_0001668
Sawtooth (st1/st2)	The process that is located along the ventral margin of the 1st valvula or the dorsal margin of the 2nd valvula	Barb, barbs [35, 165]; Cutting teeth [20]; Sägezähnchen [29, 155]; Serrula [157]; Sheath teeth [17]; Teeth [19–24]; Widerhaken [29]; Zähnchen [29]	http://purl.obolibrary.org/obo/HAO_0001681
Sclerite	The area of the cuticle that is strongly sclerotized, with thick exocuticle and is surrounded by conjunctivae	Plate [HAO]; Sclerome [HAO]	http://purl.obolibrary.org/obo/HAO_0000909
Sculpture	The area that is located on the sclerite and that is composed of repetitive anatomical structures		http://purl.obolibrary.org/obo/HAO_0000913
Segment	An anatomical structure that is metametric and is connected to other metametric subdivisions by muscles and is delimited by its sclerites		http://purl.obolibrary.org/obo/HAO_0000929
Sense organ	Multicellular anatomical structure with largely bona fide boundary that transduces some sensory stimulus to the nervous system		http://purl.obolibrary.org/obo/HAO_0000930
Sensillar patch of the 2nd valvifer (sp)	The patch that is composed of placoid sensilla adjacent to the intervalvifer articulation	Sensillar patch [HAO]; Sinneshärchen des Intervalviferengelenkes [29]	http://purl.obolibrary.org/obo/HAO_0001671
Sensillar row of the 2nd valvifer (sr)*	The row of styloconic sensilla along the proximal margin of the anterior area of the 2nd valvifer	Ramus spines [25]; Sensilla on edge of the 2nd valvifer [HAO]	http://purl.obolibrary.org/obo/HAO_0002592
Sensillum	A sense organ embedded in the integument and consisting of one or a cluster of sensory neurons and associated sensory structures, support cells and glial cells forming a single organized unit with a largely bona fide boundary		http://purl.obolibrary.org/obo/HAO_0000933
Sensillum coeloconicum (cs)	The aporous sensillum whose cuticular component is an apically rounded, hair-like structure, which is in a depression below the surface of the surrounding cuticle	Coeloconic sensillum [HAO]	http://purl.obolibrary.org/obo/HAO_0002001
Seta	The sensillum with a hair-like cuticular component	Bristle [HAO]; Hair sensillum [HAO]; Hair-like sensillum [HAO]; Sensillum trichodeum [HAO]; Sinneshärchen [29]; Trichoid sensillum [HAO]	http://purl.obolibrary.org/obo/HAO_0002299
Spermatheca*	The invagination just proximal to the vagina that accommodates sperms	Receptaculum seminis [35]; Spermathèque [50]	http://purl.obolibrary.org/obo/HAO_0000945
Sperone*	The longitudinal ridge on medially on the internal surface of the distal region of the 2nd valvula		http://purl.obolibrary.org/obo/HAO_0002590
Spine	The process that lacks non-sclerotised ring at the base		http://purl.obolibrary.org/obo/HAO_0000949
Sternite	The sclerite is located on the sternum		http://purl.obolibrary.org/obo/HAO_0000955
Sternum	The area that is located on the integument and is ventral of the ventral diaphragm		http://purl.obolibrary.org/obo/HAO_0000956
Sulcus	The groove that corresponds to a ridge	Furrow [HAO]; Suture [HAO]	http://purl.obolibrary.org/obo/HAO_0000978
T8-1st valvifer muscle	The ovipositor muscle that originates from the lateral part of female T8 and inserts on the dorsal margin of the 1st valvifer		http://purl.obolibrary.org/obo/HAO_0001640
T9-genital membrane muscle (m-T9-gm)	The ovipositor muscle that arises from the cordate apodeme and inserts dorsally on the proximal part of the genital membrane and on the opposite cordate apodeme		http://purl.obolibrary.org/obo/HAO_0001639
Tagma	The anatomical structure that is a distinctly delimited group of segments		http://purl.obolibrary.org/obo/HAO_0000988
Tendon	The portion of tissue that is fibrous, strong, composed of tendon cells and connects the muscle to the integument	~ Emergenz (am konkaven Dorsalrand des 2. Valvifers) (= tendon of the dorsal T9-2nd valvifer muscle part b) [29]	http://purl.obolibrary.org/obo/HAO_0000996
Terebra (trb)	The anatomical cluster that is composed of the 1st and 2nd valvulae	Boring apparatus and egg tube [37]; External ovipositor [32]; Inner lobes [38]; Legebohrer [29, 45, 155]; Legestachel [29]; Ovipositor [26, 27, 35, 39, 41–44, 55, 77, 79, 81, 86, 90, 93, 105, 106, 111, 151–153, 160–162, 164–166]; Ovipositor *s. str.* [29]; (Ovipositor) shaft [17–25, 71, 73, 83, 163, 174]; Shaft of ovipositor [28]; Stachel [45, 155]; Sting [36, 37, 156, 158, 159, 167]; Sting shaft [89, 168, 170, 171]; Stylus (of the ovipositor) [175]; ~ Tarière [50]	http://purl.obolibrary.org/obo/HAO_0001004
Tergite	The sclerite that is located on the tergum		http://purl.obolibrary.org/obo/HAO_0001005
Tergo-valvifer articulation (tva)	The articulation that is located between the female T9 and the 1st valvifer and is composed of the 9th tergal condyle of the 1st valvifer and the 1st valviferal fossa of the 9th tergite	Articulating process of the fulcral plate and the outer plate [35]; Tergovalvifergelenk [29]	http://purl.obolibrary.org/obo/HAO_0001636
Tergum	The area that is located on the integument and is dorsal of the ventral diaphragm	Notum [HAO]	http://purl.obolibrary.org/obo/HAO_0001006
Transvalviferal conjunctiva	The conjunctiva that transverses the 1st valvifer and separates the dorsal and ventral sclerites of the 1st valvifer		http://purl.obolibrary.org/obo/HAO_0002162
Uterus*	Anterior part of the genital chamber [173]. It is an ectodermal invagination that is the site of egg fertilization [173]. It is connected to the common oviduct anterodorsally and the vagina posteriorly [173]. It is surrounded by muscles [173]	Egg store [26]	http://purl.obolibrary.org/obo/FBbt_00004924
Vagina*	The duct that is the proximal-most region of the female genital duct, is continuous with the common oviduct and is separated from it by the gonopore	Vagin [50]	http://purl.obolibrary.org/obo/HAO_0002586
Valvillus	The sclerite that articulates on the 1st valvula and projects into the egg/poison canal	Flap [39]; Hemmplättchen [29]; Projection [176]	http://purl.obolibrary.org/obo/HAO_0001619
Venom gland*	The accessory gland that is not paired and that empties into the female reproductive duct and that is of class I gland	Acid gland [19–25, 73, 74]; Glande acide [50]; Multifid gland [73]; Poison gland [75, 89]; Sting gland [89]	http://purl.obolibrary.org/obo/HAO_0002585
Venom gland reservoir of the 2nd valvifer (vr)	The gland reservoir that is between the 2nd valvifers	Acid gland reservoir [73]; Poison reservoir [75, 89]; Reservoir (of the acid gland) [35, 37, 74]; Réservoir [50]; Venom reservoir [77, 79, 156]	http://purl.obolibrary.org/obo/HAO_0002176
Ventral	Anatomical region ventrally located on the body or body part		http://purl.obolibrary.org/obo/BSPO_0000084
Ventral 2nd valvifer-venom gland reservoir muscle (m-v-2vf-vr)*	The 2nd valvifer-venom gland reservoir muscle that originates at the medial surface of the 2nd valvifer, ventrally to the site of origin of the dorsal 2nd valvifer-venom gland reservoir muscle, and inserts laterally at the orifice of the venom gland reservoir		http://purl.obolibrary.org/obo/HAO_0002598
Ventral ramus of the 1st valvula	The area that extends external to the dorsal ramus of the 1st valvula	Ramal rod [HAO]; Ventralfortsatz [29]	http://purl.obolibrary.org/obo/HAO_0000891
Ventral ramus of the 2nd valvula	The area of the 2nd valvifer-2nd valvula-3rd valvula complex that bears the rhachis	Rami der 2. Valvulae [29]	http://purl.obolibrary.org/obo/HAO_0001107
Ventral sclerite of the 1st valvifer	The sclerite of the 1st valvifer that is ventral to the transvalviferal conjunctiva		http://purl.obolibrary.org/obo/HAO_0002164
Ventral T9-2nd valvifer muscle (m-v-T9-2vf)	The ovipositor muscle that arises from the lateral region of female T9 and inserts along the posterior part of the dorsal flange of the 2nd valvifer	Gonapophysis 8 retractor [153]; Posterior tergal muscle of the 2nd valvifer [28]; Posterior tergogonocoxal muscle [33, 34]; Retractor muscle of lancet [177]; Stylet retractor muscle [25]	http://purl.obolibrary.org/obo/HAO_0001616
Ventral wall of the 2nd valvulae (vw2)*	The ventral, usually weakly sclerotized region of the 2nd valvulae, that is medially continuous with the rachises		http://purl.obolibrary.org/obo/HAO_0002589
Vestibulum	The anatomical space that is located dorsally of abdominal sternum 7 when the latter extends beyond the abdominal sternum 8		http://purl.obolibrary.org/obo/HAO_0001082
Vitellarium*	Ovariole excluding the germarium		http://purl.obolibrary.org/obo/FBbt_00004900

The terms (abbreviations in brackets) are used and defined according to the Hymenoptera Anatomy Ontology (HAO) [52–54], the synonyms commonly found in the literature and the relevant Uniform Resource Identifier (URI) of the relevant ontologies (Hymenoptera Anatomy Ontology (HAO), Drosophila gross anatomy (FBBt), Biological Spatial Ontology (BSPO), Common Anatomy Reference Ontology (CARO); available from http://glossary.hymao.org, https://ontobee.org/, http://obofoundry.org/, respectively) are listed. Terms proposed in the present study are marked with *

## Abbreviations

1vf: 1st valvifer
1vv: 1st valvula
2vf: 2nd valvifer
2vv: 2nd valvula
3vv: 3rd valvula
au: Aulax
ba: Basal articulation
blb: Bulb
CLSM: Confocal laser scanning microscopy
co: Common oviduct
cs: Coeloconic sensillum
ct: Ctenidium
den: Dendrite
Dg: Dufour’s gland
Dgd: Dufour’s gland duct
dp2: Dorsal projection of the 2nd valvifer
dr1: Dorsal ramus of the 1st valvula
ec: Egg canal
*F*: Force
*F*_(x)_: Horizontal vector component of a force
fu: Furcula
iar: Interarticular ridge of the 1st valvifer
il1: Interlock of the 1st valvulae
iva: Intervalvifer articulation
lb: Laminated bridge
le: Lateral extensions of the 2nd valvula
LM: Light microscopy
lu2: Lumen of the 2nd valvula
*M*: Torque
m-1vf-gm: 1st valvifer-genital membrane muscle
m-a-2vf-2vv: Anterior 2nd valvifer-2nd valvula muscle
m-d-2vf-vr: Dorsal 2nd valvifer-venom gland reservoir muscle
m-d-T9-2vf-a: Dorsal T9-2nd valvifer muscle part a
m-d-T9-2vf-b: Dorsal T9-2nd valvifer muscle part b
m-p-2vf-2vv: Posterior 2nd valvifer-2nd valvula muscle
m-p-T9-2vf: Posterior T9-2nd valvifer muscle
m-T9-gm: T9-genital membrane muscle
m-v-2vf-vr: Ventral 2nd valvifer-venom gland reservoir muscle
m-v-2vf-vr-a: Ventral 2nd valvifer-venom gland reservoir muscle part a
m-v-2vf-vr-b: Ventral 2nd valvifer-venom gland reservoir muscle part b
m-v-T9-2vf: Ventral T9-2nd valvifer muscle
mb2: Median bridge of the 2nd valvifers
me: Metasoma
mr2: Medial ridge of the 2nd valvifer
oth: Olistheter
ovr: Orifice of the venom gland reservoir
prf: Post-ramus flap
rh: Rhachis
sc: Scale-like structure
SEM: Scanning electron microscopy
sp: Sensillar patch of the 2nd valvifer
sr: Sensillar row of the 2nd valvifer
SR-µCT: Synchrotron X-ray phase-contrast microtomography
st1: Sawtooth of the 1st valvula
st2: Sawtooth of the 2nd valvula
t-m-d-T9-2vf-a: Tendon of the dorsal 2nd valvifer-T9 muscle part a
T9: Female T9 (9th abdominal tergum)
TEM: Transmission electron microscope
trb: Terebra
tva: Tergo-valvifer articulation
vr: Venom gland reservoir of the 2nd valvifer
vw2: Ventral wall of the 2nd valvula
WFM: Widefield epifluorescence microscopy
